# The potential of PGPR and *Trichoderma*-based bioproducts and resistant cultivars as tools to manage clubroot disease in cruciferous crops

**DOI:** 10.3389/fpls.2023.1323530

**Published:** 2024-01-08

**Authors:** Carlos Andrés Moreno-Velandia, Francy Liliana Garcia-Arias, Lorena Dávila-Mora, Edwin Rodríguez, Alejandro Villabona-Gélvez, Eliana Gisela Revelo-Gómez, Carlos Alberto Marcillo-Paguay, Donald Heberth Riascos-Ortiz, Andrea Paola Zuluaga

**Affiliations:** ^1^ Centro de Investigación Tibaitatá, Corporación Colombiana de Investigación Agropecuaria (AGROSAVIA), Mosquera, Colombia; ^2^ Departamento de Ciencias Biológicas, Facultad de Ciencias, Universidad de los Andes, Bogotá, Colombia; ^3^ Centro de Investigación Obonuco, Corporación Colombiana de Investigación Agropecuaria (AGROSAVIA), Pasto, Colombia

**Keywords:** clubroot, biological control, host resistance, microbial consortia, *Bacillus* spp., *Trichoderma* spp.

## Abstract

The objective of this research was to determine the potential use of eco-friendly technologies to reduce the clubroot disease caused by *Plasmodiophora brassicae*, the main constraint of cruciferous crops worldwide. Two commercial bioproducts were evaluated in susceptible broccoli, one based on the PGPR consortium (*Bacillus amyloliquefaciens*, *Bacillus pumilus*, and *Agrobacterium radiobacter* K84) and the other one based on *Trichoderma koningiopsis* Th003 (Tricotec^®^ WG). Additionally, the resistant broccoli cv. Monclano® was tested under two concentrations of resting spores (RS) of *P. brassicae*, 1 × 10^3^ and 1 × 10^5^ RS g^−1^ of soil. The first phase of evaluations with broccoli was carried out under a greenhouse, while susceptible broccoli, cauliflower, and red cabbage were included in a subsequent field phase. Tebuconazole + Trifloxystrobin mixture and Fluazinam were included as positive controls. The effectiveness of the bioproducts depended on the nature of the biocontrol agent, the concentration of *P. brassicae*, and the dose of treatment. Tricotec® showed consistent plant growth promotion but no biocontrol effect against clubroot, and the rhizobacteria-based bioproduct significantly reduced the disease in both greenhouse and field experiments. Higher disease severity was observed with the higher dose of Tricotec®. Under field conditions, the rhizobacteria reduced the incidence progress by 26%, 39%, and 57% under high, medium, and low pressure of the pathogen, respectively. However, no reduction of clubroot severity under high pressure of the pathogen was observed. Complete inhibition of club formation in roots was achieved via the fungicide, but a phytotoxic effect was observed under greenhouse conditions. Fungicides reduced the incidence progress of clubroot, but not the severity under high inoculum pressure in the field. The fungicides, the bacterial treatment, and the combination of bioproducts tended to delay the progress of the disease compared with the negative control and Tricotec alone. The resistant broccoli showed a low level of disease under high concentrations of *P. brassicae* (less than 10% incidence and up to 2% severity). These results suggested the overall potential of commercial tools based on the PGPR consortium and plant resistance to control *P. brassicae*. The integration of control measures, the role of *Trichoderma* spp. in *P. brassicae*–cruciferous pathosystems, and the need to recover highly infested soils will be discussed.

## Introduction

Clubroot disease caused by the obligate pathogen *Plasmodiophora brassicae* (Protista: Phytomyxea) is the main constraint of cruciferous crops around the world (Dixon, 2014; Saharan et al., 2021). Infection by this pathogen in susceptible hosts results in the formation of galls on the roots, interrupting water and nutrient uptake, producing chlorosis in old leaves, resulting in wilting and stunting, and leading to 10%–15% yield loss (Dixon, 2006; Cao et al., 2019). Severe infestations can cause early plant death, resulting in nearly 100% yield loss (Rempel et al., 2014). This disease is difficult to control due to the complex life cycle of the pathogen, the high number of resting spores (RS) released into the soil, and its survival over time inside the host’s root cells (Cao et al., 2009).

Clubroot disease of cruciferous crops in Colombia was first reported more than 50 years ago (Torres, 1972). Vegetable production in Colombia is carried out on different types of farms, such as large agricultural grounds by moderately organized farmers whose harvested products are destined for wholesale markets; on medium-sized farms by farmers with a fixed and more specialized market; and on small plots of peasant family agriculture, where the harvested products is for self-consumption and other markets (Acevedo-Osorio and Schneider, 2020). All these areas add up to approximately 120,000 hectares (EVA, 2022), and many species of vegetables including cruciferous are grown in these areas as a monoculture, or as part of a crop rotation system. Thus, the phytosanitary limitation related to *P. brassicae* concerns not only the food production and economy of thousands of vegetable-producing families, but also the society and the environment, because of the displacement of farmers who seek new pathogen-free soils, the risk of the pathogen dispersal to these new areas, and the application of fungicides to the soil.

Cultural methods to manage this disease such as the use of limestone to increase soil pH, crop rotation, removal of symptomatic roots from the soil, disinfection protocols of agricultural tools, soil tillage equipment, and footwear have been proposed to limit pathogen dispersal and reduce pathogen inoculum in the soil (Cao et al., 2019). In addition, the use of resistant cultivars is one of the most effective, low-cost, and environmentally friendly approaches to manage this disease. Resistant cultivars can be found in all major Brassicaceae crops, but there are no resistant cultivars for all cultivated cruciferous species (Diederichsen et al., 2009). Moreover, *P. brassicae* continues to evolve new pathotypes, changing the distribution and frequency of previously existing pathotypes within individual fields. Thus, these changes in the *P. brassicae* population can overcome the resistance of previously clubroot-resistant cultivars, which result in unexpected disease outbreaks and further yield losses (Zamani-Noor et al., 2022).

The use of fungicides such as pentachloronitrobenzene (PCNB), trichlamide, flusulfamide, fluazinam and cyazofamid is one of the main strategies to prevent clubroot disease (Chai et al., 2014). Some of these molecules, also listed by Ludwig-Müller (2016), have been registered for use against *P. brassicae*. However, its use is questionable, since soil treatment with such fungicides can change soil physicochemical properties and alter soil microbial communities (Liao et al., 2022). For instance, promising fungicides such as fluazinam and cyazofamid are banned in the European Union, where farmers must use alternative control measures (Auer and Ludwig-Müller, 2023).

Thus, the use of biological control has been proposed as a potential component of an integrated disease management strategy (Cheah et al., 2000). Nevertheless, the implementation of biopesticides against clubroot at field scale is scarce despite a few studies on biocontrol against this disease. Peng et al. (2011) reported the high potential of biocontrol of clubroot by the fungus *Gliocladium catenulatum*. Bacterial strains of the genus *Bacillus* such as *Bacillus amyloliquefaciens*, *Bacillus velezensis* (Zhu et al., 2020), and *Bacillus subtilis* (Peng et al., 2011) have also helped to reduce clubroot disease. Actually, very few bioproducts have been registered in official agencies for control of clubroot such as Serenade® (US EPA, 2015) and Prestop® (US EPA, 2021).

In Colombia, cultural control of clubroot is not fully implemented and/or not completely efficient, there are no chemical options registered against this phytopathoge (ICA, 2022), and resistant cultivars for all crops are not available or do not fully meet the market requirements. For instance, the resistant hybrids of cauliflower Clapton (Syngenta, 2023a) and Clarify (Syngenta, 2023b), and the cabbage Kilazol (Syngenta, 2023c) and Tekila (Syngenta, 2023d), are resistant and tolerant to *P. brassicae* races 0, 1, and 3 respectively; the broccoli Monclano (Syngenta, 2023e) with intermediate resistance to races 0, 1, and 3 can be found in the Colombian seeds market. However, the use of these cultivars is not widely adopted, and overall, the estimated yield losses due to *P. brassicae* in the susceptible broccoli, cauliflower, and cabbage crops are approximately 43% to 73%, which depends on the inoculum pressure of the pathogen (Gómez, 2017).

Biocontrol products against clubroot in Colombia are not widely adopted, but Botero-Ramírez et al. (2015) reported that the *Trichoderma koningiopsis*-based bioproduct Tricotec® could help to reduce clubroot severity on cruciferous vegetables. Nevertheless, inconsistent effects against clubroot were also reported in the same work. Moreover, no effectiveness by Tricotec® against the Plasmodiophorida microorganism *Spongospora subterranea* reported by Mesa et al. (2017) suggested the need for additional evaluations of this bioproduct to elucidate the real potential against *P. brassicae*.

To cope with the need for clubroot disease management strategies in Colombia, we tested the integrated use of resistant varieties, two biological control products, one fungal-based Tricotec® and the other one based on a consortium of three bacterial strains (*Bacillus amyloliquefaciens, Bacillus pumilus*, and *Agrobacterium radiobacter* K84), and cultural practices in both greenhouse and field experiments. Here, we report that the use of complementary tools was successful for the management of clubroot disease.

## Materials and methods

### Plant material

Seeds of susceptible broccoli (*Brassica oleracea* var. *italica*) cv. Avenger (Sakata seeds^®^) were sown in 200-well seeding trays under commercial nursery conditions and rooted for 30 days. For the bioassays, the seedlings were planted into 600 g of soil (Andisol) and rice husk (5:1) mixture contained in black plastic bags (pots hereafter), one plant per pot. Plants were grown under unheated greenhouse conditions and watered every 3 days and fertilized once a week with the commercial nutrient solution [Merit Amarillo®, SummitAgro (N 3.6 - P_2_O_5_ 8.4 - K_2_O 7.2 and micronutrients, pH 6.9)] from 1 to 3 mL L^−1^ and 30 mL per plant.

### *P. brassicae* maintenance and inoculum preparation

*P. brassicae* was isolated from root galls of broccoli and cauliflower plants naturally infected in crops located at Gualmatán, in the municipality of Pasto (Nariño, Colombia). Because there are no studies of *P. brassicae* pathotypes in Colombia, there is no information on host specialization; thus, the inoculum was prepared from each species and kept separately. For inoculum preparation, galls were removed, washed with tap water, and disinfected with ethanol (70%) for 5 min then with sodium hypochlorite solution (5%) for 20 min followed by three washes with sterile distilled water. Then, galls were homogenized in sucrose solution (10%) in a blender following the protocol described by Fei et al. (2016). The homogenate was filtered through cheesecloth to remove root fragments, and the suspension was centrifuged at 4,000 rpm for 15 min. The pellet was resuspended in sterile deionized water, stirred, and centrifuged again. The new pellet consisting of *P. brassicae*-RS was suspended in a glycerol sterile solution (20%) and stored at −80°C until use. For soil inoculation, the concentration of RS was calculated by counting in a Neubauer chamber and the required inoculum was prepared in Tween 80 sterile solution (0.1% vol/vol). RS suspension was added by drenching (20 mL per pot) the moistened soil (water content below field capacity) 2 weeks before the transplant.

### Biocontrol agents

The commercially available bioproducts Tricotec® WG based on *T. koningiopsis* Th003 1 × 10^9^ conidia mL^−1^ and the bioproduct based on *Bacillus amyloliquefaciens* 1 × 10^8^ cfu mL^−1^, *Agrobacterium radiobacter* K84 1 × 10^8^ cfu mL^−1^, and *B. pumilus* 1 × 10^7^ cfu mL^−1^ referred to hereafter as T2 were tested as biocontrol agents against clubroot disease. Neither of the two bioproducts have a permit for use against clubroot disease. However, Tricotec® was suggested as a potential tool against clubroot (Botero-Ramírez et al., 2015) and T2 has shown high efficacy against other plant diseases (personal knowledge) and is similar to the consortium based on three bacteria, selected previously by its high efficacy against clubroot (Moreno-Velandia et al. submitted).

### Greenhouse experiments

Two experiments were carried out in an unheated greenhouse, the first one from 31 May to 21 July 2022, and the second one from 2 September to 2 November 2022. Artificial inoculation of *P. brassicae* into the soil in plastic pots was used as a model bioassay for measuring the effect of biological control agents and resistant cultivar on the clubroot development.

### Minimum effective dose of biocontrol treatments

This experiment was designed to assess if any of the tested bioproducts was effective against clubroot, and whether the efficacy depended on the dosage. Thus, four doses were tested for each bioproduct (**Table 1**). Biological treatments were applied by soil drenching, first in seedbed (5 mL per seedling), and then three times (60 mL per pot) as follows: 1 week before transplant, immediately after transplant, and 1 week after transplant. Here, treatments were challenged against the *P. brassicae* at 1 × 10^4^ RS g^−1^ of soil. Broccoli plants growing in soil inoculated with *P. brassicae* without biocontrol treatments were considered the negative control since a high level of the disease was expected in absence of control measures. Plants growing in soil free of pathogen and untreated against clubroot were considered the absolute control. Plants transplanted in soil inoculated with *P. brassicae* and treated with the fungicide Nativo® (Tebuconazole 200 g L^−1^ and Trifloxystrobin 100 g L^−1^ mixture) at a dose of 1.5 mL L^−1^ (60 mL per pot at 1 week before transplant) were included as positive control, since high reduction of the disease was expected according to previous results in a field trial (personal knowledge). A subset of broccoli plants growing in soil free of *P. brassicae*, treated with biocontrol agents under the same application scheme and dose as described above, were also included to determine whether the biocontrol treatments affected the plant growth. Plants were watered every 3 days and fertilized as described above.

**Table 1:** Effect of the bioproducts Tricotec® and T2 on plant growth and clubroot disease of broccoli under greenhouse conditions.

Treatment	Dosage	Concentration	Dry weight (g)^1^		DSI (%)
Tricotec® WG	(g L^−1^)	(conidia g^−1^)	Root	Shoot	
	0.50	5.0 × 10^5^	1.64 (0.21)	1.69 (0.08)*	96.25 (4.79) a
	0.75	7.5 × 10^5^	1.28 (0.13)	1.75 (0.07)*	93.75 (3.23) ab
	0.75	7.5 × 10^5^	1.28 (0.13)	1.75 (0.07)*	93.75 (3.23) ab
	2.00	2.0 × 10^6^	1.70 (0.15)	1.81 (0.08)*	89.38 (3.75) ab
**T2**	(mL L^−1^)	(cfu mL^−1^)			
	5.0	1.25 × 10^6^	1.05 (0.11)	1.47 (0.10)	66.25 (4.79) c
	7.5	1.88 × 10^6^	1.25 (0.16)	1.42 (0.09)	64.38 (5.91) c
	10.0	2.5 × 10^6^	1.26 (0.11)	1.37 (0.11)	64.38 (5.91) c
	20.0	5.0 × 10^6^	1.17 (0.14)	1.02 (0.08)	55.00 (0.00) c
Controls	Negative		–	–	83.75 (7.22) b
	Positive		–	–	0.00
	Absolute		1.21 (0.14)	1.37 (0.08)	0.00

^1^ Dry weight of plants was measured at 38 dat. Dry weight values are followed by the standard error in parentheses (n = 20). Asterisks indicate significant differences compared with the absolute control according to the Dunnett’s test (α = 0.05). ^2^ Disease severity index was assessed at 51 dat. The soil was inoculated with *P. brassicae* (1 × 10^4^ RS g^−1^) at 2 weeks before transplant. Average severity index values are followed by the standard deviation of data from four replicates of 10 plants per treatment. Numbers in the column with a letter in common are not significantly different according to Tukey’s test (α = 0.05). Data from positive control were not included in the analysis of biocontrol experiment because of phytotoxic effects caused by the fungicide (Tebuconazole + Trifloxystrobin).

The experimental unit of the bioassay consisted of 10 plants arranged on a base plastic tray. The bioassay was arranged under a randomized complete block design (RCBD) with four replications. The subset of plants grown in soil free of *P. brassicae* used to measure the effect of bioproducts on plant growth was sampled at 38 days after transplant (dat). Here, the entire plants were harvested, the root was carefully washed to eliminate the soil, and the root and shoot were separated and then dried in an oven for 72 h (60°C) to measure the weight of the dry biomass. On the other hand, in the experimental subset in which the pathogen was inoculated in the soil, the plants were uprooted at approximately 7 weeks after transplant and the incidence and severity of clubroot were recorded. The presence of typical galls developed by *P. brassicae* in the roots determined a plant as diseased, while the intensity of the galls, i.e., the quantity and size of the galls, was considered for recording the severity, according to the scale described by Cubeta et al. (2004).

### Effect of *P. brassicae* concentration and combination of bioproducts

To test whether the effect of biocontrol could be affected by the concentration of *P. brassicae* in the soil, and whether the combination of the treatments could improve the biocontrol efficacy, the following assay was done. Tricotec® at 2 g L^−1^ (2.0 × 10^6^ conidia mL^−1^) and 5 g L^−1^ (5.0 × 10^6^ conidia mL^−1^), T2 at 20 mL L^−1^, and the mixture of Tricotec® (2 g L^−1^) with T2 (20 mL L^−1^) were tested as biocontrol treatments. Nativo® (1.0 and 1.5 mL L^−1^) was included as positive control, but 30 mL per pot was applied by drench this time. Treatments were tested against two concentrations of *P. brassicae*, 1 × 10^3^ and 1 × 10^4^ RS g^−1^ of soil. The respective negative controls (plants growing in *P. brassicae*-inoculated soil, without treatments against clubroot) and absolute control (plants growing in soil free of pathogen and free of treatments) were also included. The treatment application scheme, the timing for the pathogen inoculation, and the plant watering and nutrition were carried out as described above. This experiment was arranged under a split plot design. The main plot was the concentration of *P. brassicae*, while the treatments for disease control accounted for the sub-plots. There were 10 plants for each experimental unit, and four replications per treatment. The incidence and severity of clubroot were recorded at the end of the eighth week after transplant as it was described above.

### Plant resistance against clubroot

To determine whether the resistant broccoli cv. Monclano® (Syngenta seeds ^©^) was resistant against *P. brassicae* isolates from Nariño, two strains isolated from cauliflower and broccoli crops, respectively, were used. Broccoli seedlings (30 days old) were transplanted into artificially inoculated soil at two pathogen concentrations: 1 × 10^3^ and 1 × 10^5^ RS g^−1^ of soil. The susceptible cultivar Avenger® was used as control. Plants of both cultivars grown in soil free of the pathogen were considered as absolute control. The trial was arranged under factorial design with a randomized complete block basic structure, with five replications per treatment. Here, the concentration of *P. brassicae*, the cultivars, and the strains of the pathogen accounted for the factors. Each experimental unit consisted of 10 pots (one plant per pot) arranged on a base plastic tray. The evaluation of clubroot was made as described above.

### Field experiments

Two set of experiments were carried out under small farm holders’ conditions at Gualmatán (Pasto, Nariño, Colombia), to test whether the performance of the treatments observed under greenhouse conditions was consistent under natural infestation of *P. brassicae.* Assays were done on the three most cultivated cruciferous species in the country: broccoli, cauliflower, and red cabbage. The first set of field experiments was performed from 29 September 2022 to 17 January 2023 and the second set was carried out from 27 April to 28 July 2023.

#### First set of field experiments

Four-week-old seedlings of red cabbage cv. Azurro F1 (Bejo seeds, Inc.), cauliflower cv. Crenique F1 (Enza Zaden Inc.), and broccoli cv. Legacy (Bayer, Seminis Inc.), all susceptible to *P. brassicae* and used by farmers in Nariño, were selected. Seedlings were grown under local commercial nursery conditions in Nariño, and bioproducts (Tricotec® and T2) were applied to the seedlings at 1 and 3 weeks after sowing (4 mL per seedling). The same volume and doses were used as described in the first experiment under greenhouse conditions, but here, we added a set of treatments in which both bioproducts were applied as a mixture for each level of dose in four treatments, i.e., Tricotec® 0.5 g L^−1^ with T2 5.0 mL L^−1^, Tricotec® 0.75 g L^−1^ with T2 7.5 mL L^−1^, Tricotec® 1 g L^−1^ with T2 10.0 mL L^−1^, and Tricotec® 2 g L^−1^ with T2 20.0 mL L^−1^.

Four-week-old seedlings were transplanted in three different farms (one species per farm) with soil naturally infested with *P. brassicae*, according to the history of clubroot disease incidence declared by the farmers. Biocontrol treatments were applied at transplant, and 1 and 3 weeks after transplant. An exclusive backpack sprayer was used for each bioproduct applying 60 mL of suspension per plant by soil drenching. The fungicide Nativo® (0.6 mL L^−1^) applied by soil drenching (30 mL plant^−1^) at transplant was included as positive control, while untreated plants were included as negative control. Agronomic practices such as fertilization and pest management were carried out following those usually practiced by the farmers.

The first set of field experiments was arranged under an RCBD with a factorial structure and four replicates per treatment. The treatments (Tricotec, T2, and Tricotec + T2) and the doses were the factors. The experimental unit consisted of one plot (2.0 × 1.8 m) with 15 plants. Plots were separated by 0.5 m and data were recorded from nine plants sampled in the center of the plot. The incidence of clubroot was recorded since the expression of external symptoms of the disease, i.e., chlorosis, wilting, and stunting, at 42, 56, 77, and 110 dat, while severity of clubroot and fresh weight of marketable cruciferous heads were recorded at harvest (110 dat).

#### Second set of field experiments

A second round of field experiments was carried out to test the consistency of the effects of Tricotec® (1 g L^−1^), T2 (20 mL L^−1^), and the mixture of these treatments, also in naturally infested soils in Gualmatán (Pasto, Nariño), and on the same cultivars of broccoli, cauliflower, and red cabbage. The treatment application scheme was the same as described above for the first set of field experiments. Since phytotoxic effects were observed with Nativo® in all previous experiments, Fluazinam (Tizca®) at a dose of 4 L ha^−1^, sprayed on the soil surface at 2 weeks before transplant, was used as positive control. Untreated plants were included as negative control. The second set of field experiments was carried out on two different farms than those used for the first experiment for red cabbage and broccoli, but cauliflower was planted in the plot where red cabbage was grown in the previous experiment. The farmers declared the incidence of clubroot in previous crops in these plots as well.

Since patches of the disease were observed in fields in the first set of experiments, square areas (2.25 m width and 2.25 m length with 14 to 15 plants) treated with each treatment were located four times throughout the experimental area, where the farmers indicated the sites with the previous presence of the disease. Thus, a completely randomized experimental design was considered for the second set of field experiments, with four replicates for each treatment. Here, the variables used for measuring the effects of the treatments were the same as described before, incidence and severity of clubroot and crop yield, on the total plants of the experimental unit.

### Data analysis

The disease severity index (DSI) was calculated with the equation DSI = [(Σ*Si*∗*Ni*)/(4∗*N*)] ∗ 100, where *Si* represents the severity grade of the symptoms, *Ni* is the number of plants in each severity grade, 4 represents the number of grades minus 1, and *N i*s the number of plants in the experimental unit (Deora et al., 2012). The area under the disease incidence progress curve (AUDPC) was calculated using the trapezoidal integration method described by Shaner and Finney (1977). The efficacy (*E*) of the treatments for reduction of the clubroot incidence, the AUDPC, and the DSI was calculated with the equation *E* = [(A−B)/A] *100, where A represents the value of the disease in the negative control and B represents the disease in the treatment under analysis.

Normality (Shapiro–Wilks, α = 0.05) and homoscedasticity (Bartlett, α = 0.05) tests were performed to the data at first. Then, one-way (for 1st) and two-way ANOVA (for 2nd and 3rd greenhouse bioassays) were performed to determine significant effects by the treatments. Data in percentage of incidence and DSI recorded in field experiments were transformed using the arcsine square root method. Two-way ANOVA was performed for the first set of field experiments while one-way ANOVA was performed for the second set of field experiments. Tukey’s test (α = 0.05) was used to compare the means of the treatments under greenhouse conditions, while Dunnett’s test was used to determine the significant effects of the treatments as compared with the negative control in field experiments. The statistical analysis was performed using the software S.A.S Enterprise (8.3 SAS Institute, Cary, NC).

## Results

### Effect of bioproducts on plant growth

Differential effects of the treatments on plant growth were evident 2 weeks after transplant of broccoli in the absence of *P. brassicae*. Leaves of plants treated with Tricotec® were bigger and greener than plants treated with the highest doses of T2 (10 and 20 mL L^−1^), and those of the absolute control (**Supplementary Figure S1**). This effect was maintained for 3 weeks more as evidenced by the dry weight of both the shoot and the root of the plants. Here, the shoot dry weight of broccoli treated with all doses of Tricotec® was significantly higher than the absolute control, while the root dry weight was significantly higher just under 1.0 g L^−1^ of Tricotec®. The dry weight of plants treated with T2 was not different from that of the absolute control, except for the shoot dry weight of plants treated with the highest dose (20 mL L^−1^), which was significantly lower (**Table 1**). Although the highest dose of T2 caused an early negative effect on plant growth, this effect was transient since the plants under this treatment were recovering the vigor as the experiment progressed as observed at 5 weeks after transplant (**Supplementary Figure S2**).

The plant growth-promoting effect exerted by Tricotec® was also evident at 2 weeks after the transplant of broccoli in soil artificially inoculated with *P. brassicae*, where treated plants were more vigorous than the rest of the treatments and the controls. By contrast, T2-treated plants showed growth decrease compared with the absolute control and no visual differences with the negative one (**Supplementary Figure S3).** However, at 5 weeks after transplant, T2-treated plants showed similar vigor to the Tricotec®-treated plants, and the negative control, but were greener than the absolute control (**Supplementary Figure S4**).

**Figure 1:**
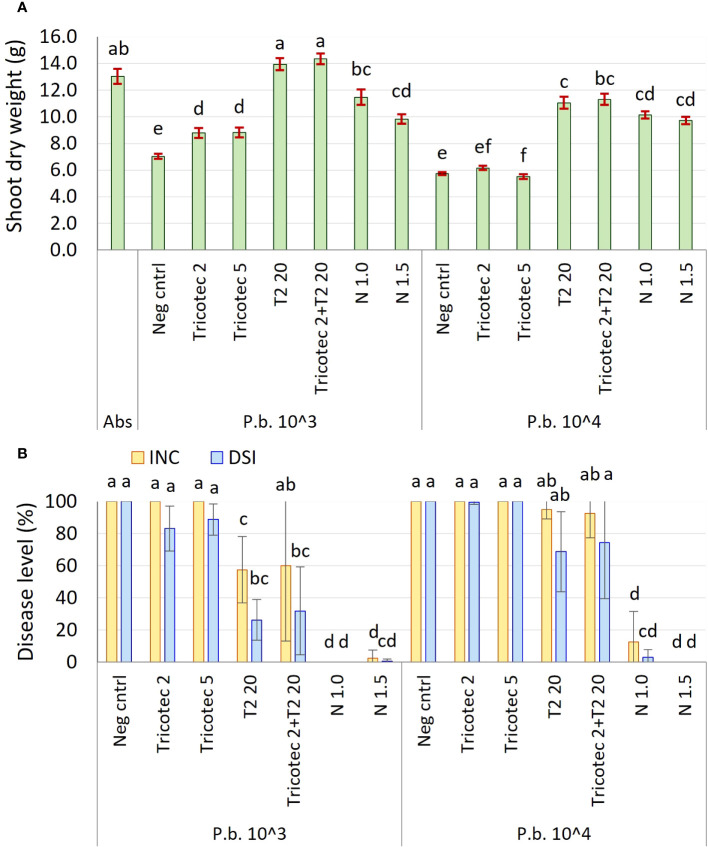
**(A)** Shoot dry weight of broccoli plants at 61 days after transplant in soil inoculated with *P. brassicae* at the concentrations of 1 × 10^3^ and 1 × 10^4^ RS g^−1^. **(B)** Effect of *P. brassicae* concentration in the soil (P.b. 1 × 10^3^ and 1 × 10^4^ RS g^−1^) on the biocontrol activity exerted by Tricotec® and T2 bioproducts. Tricotec® was applied at doses of 2 and 5 g L^−1^ (Tricotec 2 and Tricotec 5, respectively) and T2 at 20 mL L^−1^ (T2 20) alone and combined (Tricotec 2+T2 20). Scheme for bioproducts application was the same as described before. Plants grown in soil inoculated with *P. brassicae* but not treated against the disease were included as negative control (Neg cntrl) for each *P. brassicae* concentration. Plants drenched with the fungicide Nativo® at doses of 1.0 and 1.5 mL L^−1^ were considered as positive controls (N 1.0 and N 1.5, respectively). Plants grown in soil free of both pathogen and treatments were included as absolute control (Abs). Bars on the top of the shoot dry weight columns represent the standard error of the data (*n* = 40). Treatments sharing the same letter are not significantly different according to the Tukey test (α = 0.05). Bars on the disease columns represent the standard deviation of data (*n* = 4). The columns from the same variable (INC: Incidence, DSI: Disease severity index) sharing the same letter are not significantly different according to Tukey test (α = 0.05).

The second experiment under greenhouse conditions showed that the shoot dry weight of plants grown in soil artificially inoculated with *P. brassicae* and treated with bioproducts was significantly affected by the concentration of the pathogen at 61 dat, where the higher concentration of *P. brassicae* caused the lower aerial dry weight. The lowest dry weight values were shown by plants treated with both doses of Tricotec® (2 and 5 g L^−1^) and the negative control, under both concentrations of the pathogen in the soil (**Figure 1A**). In contrast, the shoot dry weight of plants treated with T2 was like the absolute control under the low concentration of the pathogen (1 × 10^3^ RS g^−1^), and it was slightly lower than the control under a high concentration of the pathogen (1 × 10^4^ RS g^−1^). The combination of Tricotec® and T2 did not show improved plant growth as compared with the individual application of T2. Shoot dry weight of plants treated with the fungicide was similar under both doses and both concentrations of *P. brassicae* in the soil, but was lower than the absolute control, showing phytotoxic effects of the fungicide on the plants under these experimental conditions (**Figure 1A**).

### Effect of bioproducts on clubroot development

The bioassay in which individual applications of four doses of the bioproducts Tricotec® and T2 were evaluated against clubroot showed no reduction of the disease incidence by the treatments. Moreover, none of the tested doses of Tricotec® reduced the clubroot DSI as compared with the negative control (DSI = 84%). However, the DSI was significantly lower in plants treated with T2 at all tested doses compared with the negative control, but no differences were found among the T2 doses (**Table 1**). T2 reduced the DSI from 21% to 34%. Although plants treated with Nativo® did not develop clubbed roots, plant shoots did not develop well, showing phytotoxicity symptoms.

Based on these results, we hypothesized that a better control of clubroot could be achieved by a higher dose of Tricotec® or by the combination of both bioproducts (Tricotec + T2), and under lower pathogen inoculum pressure. Thus, the second experiment under greenhouse conditions carried out to test this hypothesis showed that neither the low dose nor the high dose of Tricotec® reduced the clubroot severity under high and low concentration of *P. brassicae* in the soil. In contrast, T2 significantly reduced the disease severity under both concentrations of the pathogen in the soil, having higher efficacy under the lower pathogen pressure. Similar results were found in plants treated with the combination of Tricotec® and T2, but it is likely that the effect was exerted by T2. Lastly, plants treated with both doses of the fungicide showed the lowest disease incidence (less than 12%) and severity (less than 0.5%), but the low shoot dry weight values of these plants depended on the phytotoxic effects when it is applied by drench (**Figure 1B**).

### Plant resistance against clubroot

**Figure 2:**
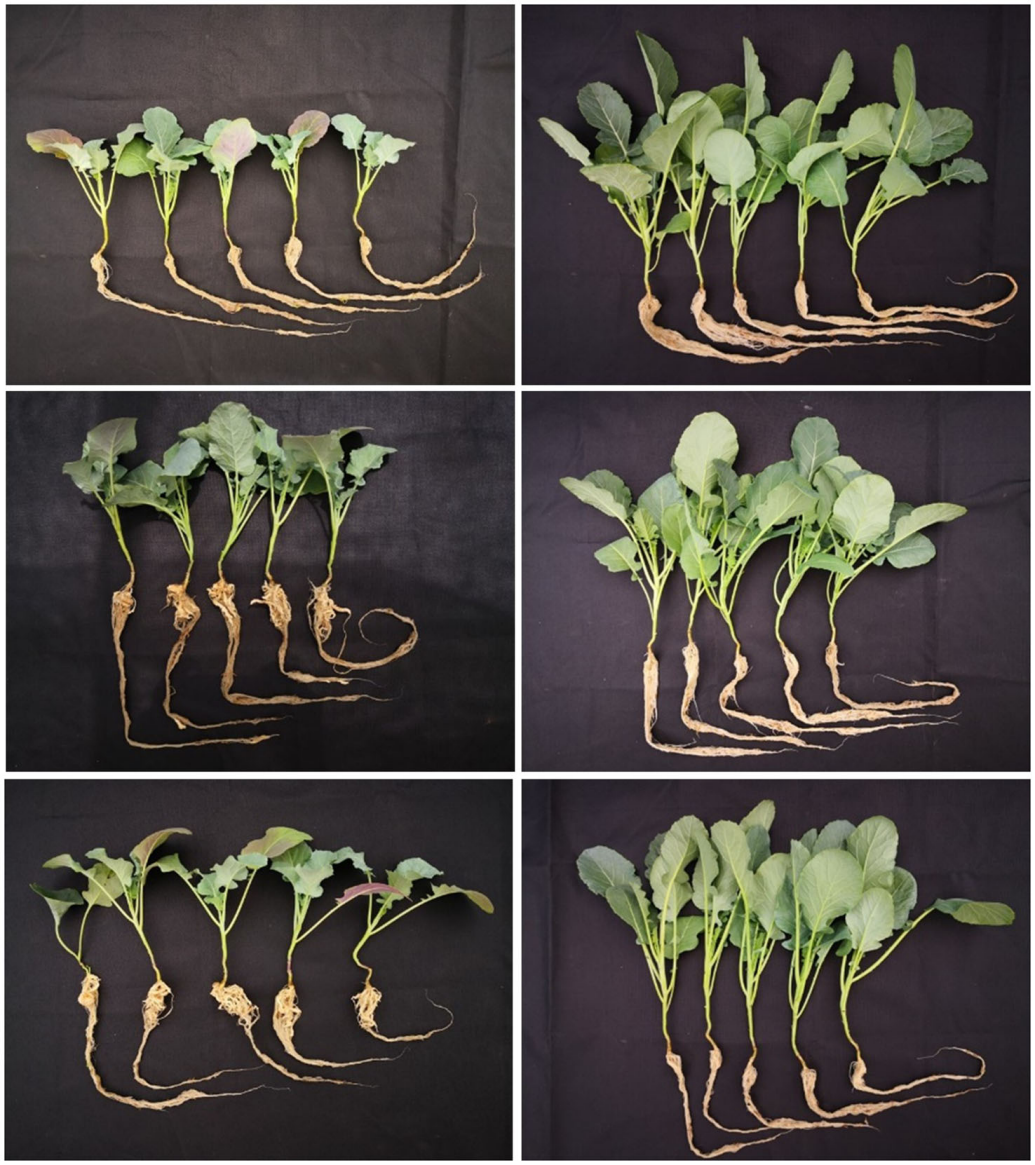
Clubroot symptoms on the susceptible broccoli cv. Avenger (left panel) and the resistant cv. Monclano (right panel) 7 weeks after transplant in soil inoculated with low (1 × 10^3^ RE g^−1^, middle row) and high (1 × 10^5^ RE g^−1^, bottom row) concentrations of *P. brassicae* (inoculum from broccoli plants). Avenger and Monclano controls in the top row.

This experiment showed no differences between the *P. brassicae* inoculum origin on the disease development. Both strains (isolated from cauliflower and broccoli) caused the same disease severity in broccoli. Clubroot reached a severity of 100% in the susceptible cv. Avenger grown under both low and high concentrations of *P. brassicae*, while the disease incidence on the resistant cv. Monclano was up to 2% and 8% under the low and the high concentrations of the pathogen, respectively. The DSI was not significantly different between the concentrations of *P. brassicae*, and it was from 75% to 86% on the susceptible cv. Avenger, while symptomatic plants in the resistant cv. Monclano showed up to 2% of DSI (**Figure 2**).

### Clubroot development in field experiments

**Figure 3:**
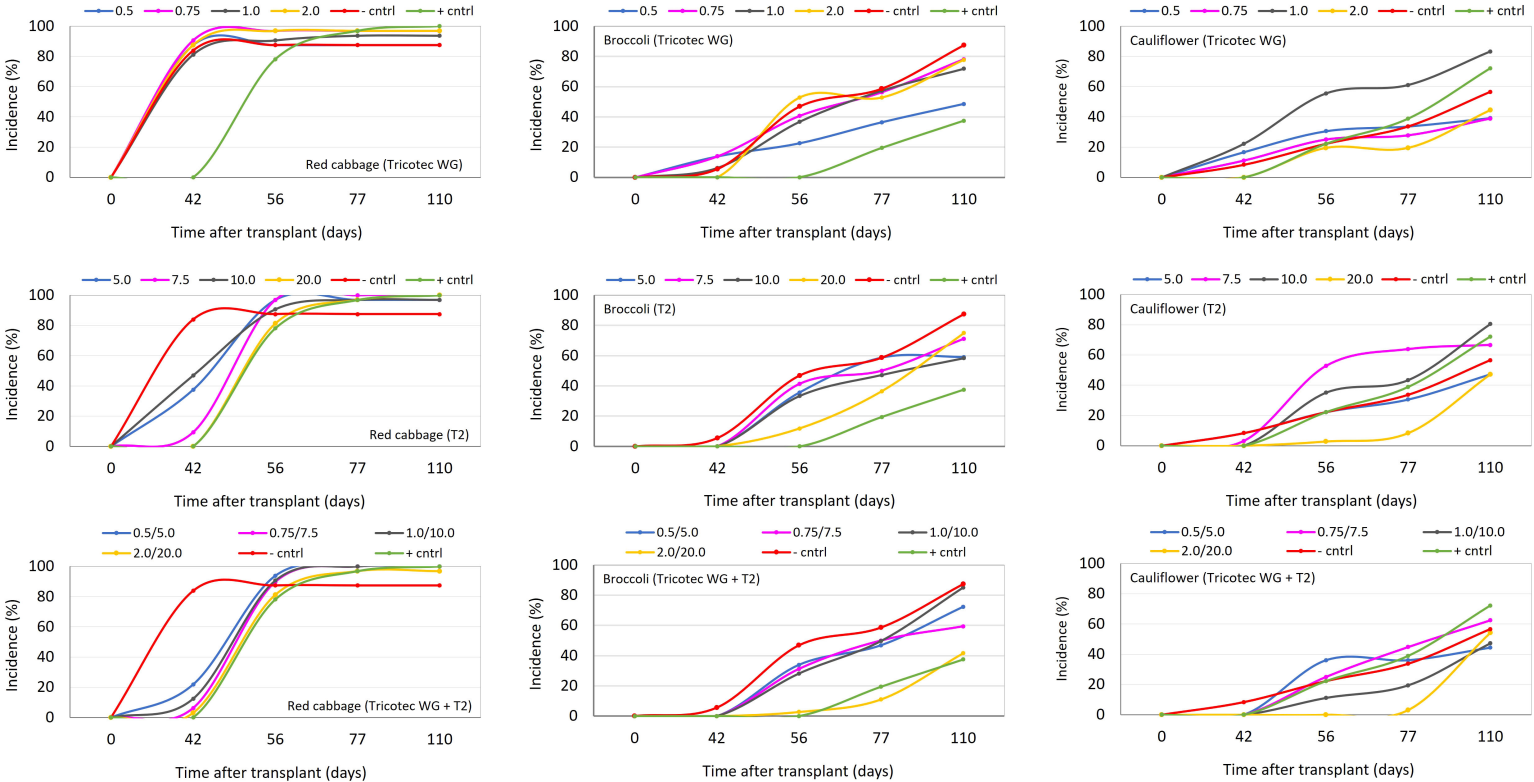
Clubroot incidence progress curves in red cabbage (graphics in left column), broccoli (graphics in central column), and cauliflower (graphics in right column) crops under the effect of different doses of the biocontrol treatments Tricotec® (top row), T2 (central row), and the combination of them (bottom row) in the first set of field experiments. Tested doses of Tricotec® were 0.5, 0.75, 1.0, and 2.0 g L^−1^. Tested doses of T2 were 5.0, 7.5, 10.0, and 20.0 mL L^−1^. In the negative control (− cntrl), the plants were untreated against clubroot. The positive control (+ cntrl) consisted of drench application of the fungicide Nativo® (0.6 mL L^−1^, 30 mL plant^−1^) at transplant.

In the first set of field experiments, we wanted to test the same doses of Tricotec® and T2 described above for the first experiment under greenhouse, but here the combination of both bioproducts at the respective doses were included. According to the level of the disease observed in the three experimental areas, these soils were classified as highly, moderately, and slightly infested with *P. brassicae*, for red cabbage, broccoli, and cauliflower plots, respectively. The results here showed consistency with observations made under greenhouse. Thus, in the plot with high disease pressure, clubroot in red cabbage was uniform, and T2-based treatments, the fungicide, and all doses of the combination of Tricotec® with T2 delayed the incidence progress of clubroot (**Figure 3**). However, none of the treatments reduced the final value of incidence or the DSI (**Table 2**). Nevertheless, T2 (10 and 20 mL L^−1^) and the highest dose of the combination of Tricotec® with T2 delayed the expression of clubroot symptoms for more than 42 dat (**Figure 3**) and allowed the highest yields (**Table 2**).

**Table 2:** Effect of the bioproducts Tricotec® and T2 on clubroot development and yield of cruciferous crops in the first set of field experiments.

Treatment	Dose	*First set of field experiments*							
		*Red cabbage (High infestation of P. brassicae into the soil)*							
**Tricotec® WG**	(g L^−1^)	Inc t_i_ (%)	Inc t_f_ (%)	AUDPC (% × days)	DSI (%)	Efficacy/ Inc t_f_ (%)	Efficacy/ AUDPC (%)	Efficacy/ DSI (%)	Yield (tons ha^−1^)
	0.5	87.5 (10.2)	87.5 (10.2)	7,787.5 (908.4)	87.5 (10.2)	0.00	−1.3	0.00	0.6 (0.4)
	0.75	90.6 (6.3)	96.9 (6.3)	8,446.9 (469.5)	96.9 (6.3)	−10.7	−9.9	−10.7	0.7 (0.4)
	1.0	81.3 (7.2)	93.8 (12.5)	7,939.0 (884.3)	100.0 (0.0) *	−7.1	−3.3	−14.3	0.9 (0.7)
	2.0	87.5 (0.0)	96.9 (6.3)	8,359.4 (381.3)	96.9 (6.3)	−10.7	−8.7	−10.7	0.5 (0.2)
**T2**	(mL L^−1^)								
	5.0	37.5 (32.3) *	96.9 (6.3)	6,959.4 (885.5)	96.9 (6.3)	−10.7	9.5	−10.7	1.5 (0.5)
	7.5	9.4 (12.0) *	100.0 (0.0)	6,307.8 (403.2)	100.0 (0.0) *	−14.3	17.9	−14.3	4.1 (1.6) *
	10.0	46.9 (31.3)	96.9 (6.39)	7,112.5 (836.2)	96.9 (6.3)	−10.7	7.5	−10.7	4.6 (1.9) *
	20.0	0.0 (0.0) *	100.0 (0.0)	5,687.5 (426.7) *	99.2 (1.6)	−14.3	26.0	−13.4	8.2 (3.7) *
**Tricotec® WG + T2**	(g L^−1^ + mL L^−1^)								
	0.5 + 5.0	21.9 (21.3) *	100.0 (0.0)	6,603.1 (707.7)	100.0 (0.0) *	−14.3	14.1	−14.3	2.1 (1.3)
	0.75 + 7.5	6.3 (7.2) *	100.0 (0.0)	6,095.3 (360.4)	100.0 (0.0) *	−14.3	20.7	−14.3	3.5 (0.8) *
	1.0 + 10.0	12.5 (25.0) *	100.0 (0.0)	6,285.9 (866.5)	99.2 (1.6)	−14.3	18.2	−13.4	4.6 (1.6) *
	2.0 + 20.0	3.1 (6.3) *	96.9 (6.3)	5,723.4 (516.2)	96.9 (6.3)	−10.7	25.6	−10.7	8.8 (1.8) *
**Control**	Positive	0.00 (0.0) *	100.0 (0.0)	5,632.8 (444.6)	100.0 (0.0) *	−14.3	26.7	−14.3	3.7 (1.8) *
	Negative	83.9 (6.0)	87.5 (10.2)	7,687.5 (753.9)	87.5 (10.2)				0.6 (0.3)
		*Broccoli (Moderate infestation of P. brassicae into the soil)*							
**Tricotec® WG**	(g L^−1^)	Inc t_i_ (%)	Inc t_f_ (%)	AUDPC (% × days)	DSI (%)	Efficacy/ Inc t_f_ (%)	Efficacy/ AUDPC (%)	Efficacy/ DSI (%)	Yield (tons ha^−1^)
	0.5	13.9 (27.8)	48.6 (40.9)	2,570.3 (3,212.2)	36.0 (40.74)	44.4	35.8	49.8	21.1 (5.5)
	0.75	13.9 (27.8)	78.1 (43.8)	3,907.6 (3,125.8)	65.9 (39.87)	10.7	2.4	8.1	19.0 (1.3)
	1.0	6.3 (12.5)	71.9 (48.3)	3,551.9 (2,783.8)	56.4 (41.5)	17.9	11.3	21.5	22.9 (2.2)
	2.0	0.0 (0.0)	77.8 (37.4)	3,631.9 (2,173.4)	71.9 (37.7)	11.1	9.3	−0.2	15.6 (6.3)
**T2**	(mL L^−1^)								
	5.0	0.0 (0.0)	59.1 (39.5)	3,186.3 (2,109.0)	36.5 (31.8)	32.4	20.4	49.2	20.9 (4.2)
	7.5	0.0 (0.0)	71.2 (19.8)	3,247.6 (1,550.0)	47.3 (14.9)	18.6	18.9	34.1	20.4 (3.5)
	10.0	0.0 (0.0)	58.3 (50.0)	2,820.8 (2,961.6)	47.2 (47.2)	33.3	29.6	34.2	20.3 (0.9)
	20.0	0.0 (0.0)	75.0 (42.1)	2,428.5 (1,491.0)	48.9 (26.8	14.3	39.4	31.8	24.1 (4.2)
**Tricotec® WG + T2**	(g L^−1^ + mL L^−1^)								
	0.5 + 5.0	0.0 (0.0)	72.3 (21.1)	3,052.7 (1,912.1)	51.3 (22.5)	17.4	23.8	28.5	20.2 (4.2)
	0.75 + 7.5	0.0 (0.0)	59.4 (47.2)	2,876.6 (2,822.1)	42.2 (44.3)	32.1	28.2	41.2	23.0 (5.1)
	1.0 + 10.0	0.0 (0.0)	85.1 (18.3)	3,236.5 (2,071.9)	63.5 (26.9)	2.8	19.2	11.6	17.9 (3.7)
	2.0 + 20.0	0.0 (0.0)	41.7 (50.0)	1,036.1 (1,205.2)	30.1 (26.1)	52.4	74.1	58.1	17.8 (4.7)
**Control**	Positive	0.0 (0.0)	37.5 (40.4)	1,143.8 (1,455.2)	19.4 (20.9)	57.1	71.4	72.9	13.4 (3.4)
	Negative	5.6 (11.1)	87.5 (17.7)	4,003.9 (1,698.6)	71.8 (27.6)				19.5 (3.4)
		*Cauliflower (Low infestation of P. brassicae into the soil)*							
**Tricotec® WG**	(g L^−1^)	Inc t_i_ (%)	Inc t_f_ (%)	AUDPC (% × days)	DSI (%)	Efficacy/ Inc t_f_ (%)	Efficacy/ AUDPC (%)	Efficacy/ DSI (%)	Yield (tons ha^−1^)
	0.5	16.7 (33.3)	39.2 (40.8)	2,558.2 (3,631.3)	29.3 (45.3)	30.7	−3.7	21.6	53.6 (4.2)
	0.75	11.1 (22.2)	38.9 (41.1)	2,140.3 (3,352.1)	45.8 (45.7)	31.3	13.2	−22.5	52.5 (12.0)
	1.0	22.2 (25.7)	83.3 (33.3)	4,619.4 (2,846.2)	67.4 (36.8)	−47.2	−87.4	−80.1	28.2 (12.6)
	2.0	0.0 (0.0)	44.4 (37.4)	1,598.6 (1,544.9)	18.1 (14.4)	21.5	35.2	51.7	54.0 (9.2)
**T2**	(mL L^−1^)								
	5.0	8.3 (16.7)	47.2 (49.2)	2,226.4 (2,532.0)	30.6 (35.9)	16.6	9.7	18.3	54.4 (5.6)
	7.5	3.1 (6.3)	66.7 (47.1)	3,836.1 (2,743.8)	57.6 (44.3)	−17.8	−55.6	−54.1	45.6 (10.1)
	10.0	0.0 (0.0)	80.6 (19.0)	3,114.8 (2,008.8)	54.4 (24.5)	−42.3	−26.3	−45.5	47.7 (13.1)
	20.0	0.0 (0.0)	47.2 (49.2)	1,052.8 (1,128.9)	22.9 (25.0)	16.6	57.3	38.8	44.7 (7.8)
**Tricotec® WG + T2**	(g L^−1^ + mL L^−1^)								
	0.5 + 5.0	0.0 (0.0)	44.4 (45.4)	2,340.3 (2,685.2)	26.4 (29.4)	21.5	5.0	29.5	58.6 (3.2)
	0.75 + 7.5	0.0 (0.0)	62.5 (43.3)	2,678.1 (2,425.6)	37.9 (36.3)	−10.4	−8.6	−1.4	51.4 (10.3)
	1.0 + 10.0	0.0 (0.0)	47.2 (41.0)	1,498.6 (1,568.8)	25.7 (35.2)	16.6	39.2	31.3	51.4 (13.9)
	2.0 + 20.0	0.0 (0.0)	54.2 (32.8)	978.1 (553.5)	21.9 (12.4)	4.3	60.3	41.5	57.6 (5.9)
**Control**	Positive	0.0 (0.0)	72.2 (19.2)	2,630.6 (1,728.8)	34.6 (31.9)	−27.6	−6.7	7.4	42.9 (5.0)
	Negative	8.3 (16.7)	56.6 (34.3)	2,465.5 (2,341.6)	37.4 (18.4)				40.6 (10.4)

Inc t_i_: initial mean value of clubroot incidence recorded. Inc t_f_: mean value of clubroot incidence recorded at the end of the experiment. AUDPC: area under disease incidence progress curve. DSI: Disease severity index. Efficacy/IC t_f_: efficacy of the treatments to reduce the final incidence of clubroot. Efficacy AUDPC: efficacy of treatments to reduce the progress of the clubroot incidence. Efficacy DSI: Efficacy of treatments to reduce the severity of clubroot at the end of the experiment. The numbers in parentheses represent the standard deviation of data (n = 4). The efficacies were calculated with the mean value of each variable. The asterisk indicates significant differences of the treatment compared with the negative control according to Dunnett’s test (α = 0.05).

In plots with moderate and low infestation of *P. brassicae*, we can describe the results only as trends because of the high variability of the response, most likely due to the patchy distribution of the pathogen in the soil. Thus, under moderate infestation of *P. brassicae* into the soil, the lowest dose of Tricotec® (0.5 g L^−1^), the highest dose of T2 (20 mL L^−1^), and the mixture of both bioproducts (at the highest dose) delayed the expression of clubroot symptoms in broccoli (up to 56 dat). The incidence of the disease at 56 dat under these treatments reached 23%, 12%, and 3%, respectively, and the DSI was 36%, 49%, and 30%, respectively, at the end of the experiment.

The average yield of broccoli was high under Tricotec 0.5 g L^−1^ (21 tons ha^−1^), T2 20 mL L^−1^ (24 tons ha^−1^), and the mixture of both at 0.75 g L^−1 + ^7.5 mL L^−1^ (23 tons ha^−1^) (**Figure 3**; **Table 2**). Finally, under low pressure of *P. brassicae*, it was observed that the highest dose of the three biocontrol treatments (Tricotec 2 g L^−1^, T2 20 mL L^−1^, and the mixture 2 g L^−1 + ^20 mL L^−1^) delayed the expression of clubroot symptoms in cauliflower (>42, 56, and 77 dat, respectively) (**Figure 3**), with incidences of 22%, 3%, and 3%, respectively; the DSI was 18%, 23%and 22%, respectively. The yield of cauliflower under these treatments was 54, 45, and 58 tons ha^−1^, respectively (**Table 2**).

Based on the results from the first set of experiments undertaken under field conditions, T2 at a dose of 20 mL L^−1^ was selected because of both the high and consistent efficacy to control clubroot. This treatment was compared with Tricotec® (1 g L^−1^) and with the combination of them, mixed in the same suspension for the applications. For this second set, the cauliflower crop was established in the plot where red cabbage was grown in the first experimental set, while the new red cabbage and broccoli crops were established in different plots than the areas used in the first field experiment.

According to the clubroot incidence, the crops in the second experimental phase were also grown under high (cauliflower plot), moderate (red cabbage), and low (broccoli) pressure of pathogenic inoculum (**Supplementary Figure S5**). It was observed that the fungicide (Tizca® SC, Fluazinam, FMC^©^) was the most efficient treatment delaying the appearance of symptomatic plants, up to 49 and 62 dat, under high and moderate infestation, respectively, while the fungicide and T2 treatments delayed the expression of the symptoms for more than 83 dat under the low-infestation condition (**Supplementary Figure S5**; **Table 3**). Under the high-infestation condition (cauliflower crop), neither of the treatments reduced the incidence or the DSI, and the yield loss was 100%. Under moderate infestation (red cabbage crop), Fluazinam was the most efficient treatment, but Tricotec® and the mixture of both biologicals reduced the progress of the disease by approximately 50% compared to the negative control (**Table 3**). However, all the biological treatments showed similar effects at the end of the season, with efficacy of incidence reduction by 53%, 32%, and 45% for Tricotec®, T2, and the mixture of both, respectively, and a reduction of the DSI by 24%, 17%, and 14%, respectively (**Table 3**). Finally, under low-infestation conditions (broccoli crop), in addition to chemical treatment, just T2 reduced both the incidence and DSI by 82% and 91%, respectively. Regarding the yield in red cabbage (moderate infestation), this was too low because of the clubroot, and similar for all treatments, ranging from 6 to 13 tons ha^−1^. On the other hand, the yields of broccoli (low infestation) were close to the commercial ones, ranging from 48 to 55 tons ha^−1^, but no differences among treatments were detected (**Table 3**).

**Table 3:** Effect of the bioproducts Tricotec® and T2 on clubroot development and yield of cruciferous crops in the second set of field experiments.

Treatment	*Second set of field experiments*									
	*Cauliflower (High infestation of P. brassicae into the soil)*									
	Inc t_i_ (%)	Inc t_f_ (%)	AUDPC (% × days)	DSI (%)	Efficacy/ Inc t_f_ (%)	Efficacy/ AUDPC (%)		Efficacy/ DSI (%)		Yield (ton ha^−1^)
Tricotec 1 g L^−1^	94.6 (6.8)	100.0 (0.0)	6,322.3 (263.1)	100.0 (0.0)	0.0	−11.3		0.0		0.0
T2 20 mL L^−1^	94.6 (10.7)	100.0 (0.0)	6,322.3 (455.4)	100.0 (0.0)	0.0	−11.3		0.0		0.0
Tricotec + T2	98.2 (3.6)	100.0 (0.0)	6,494.6 (110.7)	100.0 (0.0)	0.0	−14.3		0.0		0.0
Fluazinam	32.1 (45.4) *	100.0 (0.0)	4,446.4 (1,406.3)	100.0 (0.0)	0.0	21.7		0.0		0.0
Negative control	82.1 (31.1)	100.0 (0.0)	5,680.4 (1,444.3)	100.0 (0.0)						0.0
	*Red cabbage (Moderate infestation of P. brassicae into the soil)*									
Tricotec 1 g L^−1^	6.7 (9.4)	30.0 (13.9)	1,219.2 (565.2)	69.6 (7.9)	52.6	50.6		24.1		13.3 (7.3)
T2 20 mL L^−1^	23.3 (20.7)	43.3 (32.9)	2,218.3 (1,685.5)	75.8 (26.0)	31.6	10.1		17.3		10.1 (3.2)
Tricotec + T2	5.0 (10.0)	35.0 (20.6)	1,043.3 (700.1)	78.8 (16.8)	44.7	57.7		14.1		8.6 (5.3)
Fluazinam	0.0 (0.0)	11.7 (8.4) *	265.0 (305.8) *	59.2 (9.9) *	81.6	89.3		35.5		11.8 (1.6)
Negative control	15.0 (14.8)	63.3 (16.8)	2,468.3 (950.8)	91.7 (4.9)						10.7 (5.9)
	*Broccoli (Low infestation of P. brassicae into the soil)*									
Tricotec 1 g L^−1^	5.0 (10.0)	53.3 (54.2)	1,894.2 (2,100.0)	43.3 (48.2)	−45.5	−157.4		−85.7		50.4 (11.5)
T2 20 mL L^−1^	0.0 (0.0)	6.7 (5.4)	138.3 (218.0)	2.1 (2.1)	81.8	81.2		91.1		50.6 (4.9)
Tricotec + T2	0.0 (0.0)	40.0 (30.3)	307.5 (226.2)	22.5 (17.8)	−9.1	58.2		3.6		54.9 (3.0)
Fluazinam	0.0 (0.0)	11.7 (19.1)	71.7 (78.8)	4.2 (7.3)	68.2	90.3		82.1		54.7 (5.2)
Negative control	5.0 (10.0)	36.7 (32.0)	735.8 (1,065.2)	23.3 (22.2)						47.7 (6.5)

Inc t_i_: initial mean value of clubroot incidence recorded. Inc t_f_: mean value of clubroot incidence recorded at the end of the experiment. AUDPC: area under disease incidence progress curve. DSI: Disease severity index. Efficacy/IC t_f_: efficacy of the treatments to reduce the final incidence of clubroot. Efficacy AUDPC: efficacy of treatments to reduce the progress of the clubroot incidence. Efficacy DSI: Efficacy of treatments to reduce the severity of clubroot at the end of the experiment. The numbers in parentheses represent the standard deviation of data (n = 4). The efficacies were calculated with the mean value of each variable.

## Discussion

The scarce implementation of cultural practices against *P. brassicae*, the increasing presence of the disease in all places where crucifers are grown, and the lack of registered biological control agents have led us to search for biological-based alternatives to control clubroot. In this way, screening of native strains of fungi and bacteria against clubroot was carried out previously. As a result, the consortium based on *Pseudomonas fluorescens* Ps006, *Lysinibacillus xylanilyticus* Br042, and *Bacillus velezensis* Bs006 was selected as the most efficient treatment (Moreno-Velandia et al. submitted). Available local bioproducts were also tested in this work. Thus, the bacterial consortium (*B. amyloliquefaciens*, *B. pumilus*, and *A. radiobacter*)-based bioproduct (T2) and Tricotec® (*T. koningiopsis* Th003) were considered for this research since they have shown high effectiveness against soil-borne phytopathogens (Moreno-Velandia et al., 2009; Cruz-Barrera et al., 2022; Vargas-Baquero and Cotes, 2023).

Interestingly, the two distinct active ingredients of these bioproducts showed contrasting effects on both plant growth promotion and plant protection against *P. brassicae*. On one hand, *Trichoderma*-based bioproduct effectively promoted the plant growth of broccoli in soil free of *P. brassicae*; on the other hand, the rhizobacteria-based bioproduct reduced the plant growth. However, when *P. brassicae* was added to the soil, Tricotec® showed just a transient plant growth promotion effect and was ineffective to control clubroot, while the plant growth reduction effect shown by T2 was temporary and showed a high plant protection effect in plants growing in soil infested with *P. brassicae*.

Although the amount of research on biocontrol of *P. brassicae*–cruciferous is not as vast as in other soil-borne pathosystems, the increasing scientific literature on this area shows the interest and the potential of this method against clubroot (Auer and Ludwig-Müller, 2023). Since some fungicides with demonstrated efficacy to control clubroot are banned in the European Union, and there is interest in developing sustainable clubroot management around the world, further work based on the combination of crop management practices, resistant cultivars, and biological control approaches must be carried out. According to the review made by Auer and Ludwig-Müller (2023), the first indexed publications in scientific literature on biocontrol treatments against clubroot disease are from the late 1990s. *Cladophialophora chaetospira* (Narisawa et al., 1998), *Phoma glomerata*, and *Phoma wasabiae* by Arie et al. (1998) and various species of *Trichoderma* by Cheah and Page (1997) and Cheah et al. (2000) were the first species of fungi tested as potential antagonists against clubroot under greenhouse and field conditions. On the other hand, the history of the evaluations of bacterial species controlling clubroot goes back to the early 1990s. The potential of *Pseudomonas* spp. as biocontrol agents against clubroot was reported by Elsherif and Grossman (1991). Bhattacharya and Pramanik (1998); Cheah et al. (2000), and Lee et al. (2008) indicated that some species of actinomycetes may be useful biocontrol agents for the control of clubroot. Some species of *Bacillus* and *Lysobacter* genera have also shown biocontrol effects against clubroot (Ahmed et al., 2020). Moreover, evaluations of microbial consortia on clubroot have also been reported (Kurowski et al., 2009; Guo et al., 2019).

*Trichoderma* is not only recognized as the main biocontrol agent against soil-borne pathogens, but also promotes plant growth, improves the efficiency of nutrient use, and enhances plant resistance (Sood et al., 2020). Many species of *Trichoderma* establish an effective plant protection relationship with the host plants through complex and interrelated mechanisms such as stimulation of plant defense/resistance against biotic and abiotic stresses, mycoparasitism, antibiosis, competition, nutrient solubilization, and the production of phytohormones and other plant growth promoters (El Enshasy et al., 2020). However, the mechanisms underlying the activity of *Trichoderma* spp. on clubroot are not clear at all, even more so considering that success stories are scarce.

A low number of *Trichoderma* species have been tested against *P. brassicae*, and in some works, the species were not reported. From the review by Auer and Ludwig-Müller (2023), just a few species have been tested, mainly *T. harzianum* followed by *T. virens*, *T. asperellum*, and *T. koningiopsis*. Moreover, no studies on modes of action by the biocontrol agents against *P. brassicae* were done, with the exception of that described in Yu et al. (2015), where the inhibition of RS germination by *T. harzianum* T4 filtrates was demonstrated, suggesting an antibiosis effect, in addition to the regulation of the microbial communities in the rhizosphere that could influence *P. brassicae* growth. Li et al. (2020) associated the biocontrol effect of *T. harzianum* against clubroot with its colonization of the rhizosphere and the subsequent change of fungal microbial community, including 18 plant pathogens. On the other hand, the modes of action of the tested strains of *Bacillus* spp. against *P. brassicae* have also been related to the production of antimicrobial metabolites (Peng et al., 2011). However, it is worth highlighting that, as far as we know, no studies to determine the specific modes of action by biocontrol agents against *P. brassicae*/clubroot disease have been done.

This work shows both the positive effects of a well-known member from the beneficial *Trichoderma* genus on plant growth and no effect on biocontrol, and the negative effects of a new bacterial consortium on plant growth and positive effects on biocontrol. In both cases, this work calls to thoroughly study the interactions between *T. koningiopsis* Th003 or *Trichoderma* spp. with both cruciferous species and *P. brassicae* resulting in the stimulation of clubroot development, as well as the study of the mechanisms of action exerted by the bacterial consortium influencing the reduction of the clubroot disease.

It is interesting to see, from the work of Auer and Ludwig-Müller (2023), the high variability of the biocontrol effect by each group of beneficial microorganisms, and even more those cases in which clubroot development was favored under certain biological treatments, including *Trichoderma*. Moreover, studies that elucidate why the biocontrol agents promote disease are not usual, but those should be executed to further understand the complex biocontrol agent–plant–pathogen interactions. In this work, the first bioassay under greenhouse conditions led us to hypothesize that a higher dose of Tricotec® was necessary to increase the efficacy. However, contrary to what we expected, a greater severity with the higher dose of Tricotec® was found. These results led us to propose new hypotheses that deserve to be tested through new research. For example, it would be relevant to test whether the biocontrol of clubroot is ruled under a strain/species-specific interaction between *Trichoderma* and the pathogen. In addition, it would be interesting to determine the role of phytohormone production by *Trichoderma* spp. strains on clubroot biocontrol.

One of the alternatives to overcome the variability of biocontrol of plant diseases has been the application of beneficial microorganisms in consortia (Izquierdo-García et al., 2020). Interestingly, the bioproduct based on the consortium of *B. amyloliquefaciens*, *B. pumilus*, and *A. radiobacter* K84 showed high and consistent efficacy controlling clubroot in this study, both in the greenhouse and in the field experiments. Further experiments should be carried out to evaluate the individual contributions of these strains to the biocontrol or determine whether the effect is the result of the tri-partite effect, under an additive or synergistic interaction. Considering that the T2 bioproduct contains two strains of the *Bacillus* genus, their action against *P. brassicae* could probably be related to its ability to destroy the cell walls of RS, inhibiting the early infection of root hairs and reducing the differentiation of primary plasmodia and the formation of secondary zoosporangia of *P. brassicae* through antibiotics such as fengycins and chitinase-like proteins such as PBT1, as reported previously (Li, 2013; Zhu et al., 2020; He et al., 2023). However, additional experimentation should be done to study the specific modes of action exerted by the efficient consortium in this work against *P. brassicae*, and its interaction with the cruciferous plants.

We followed a research scheme from semi-controlled conditions in greenhouse to the uncontrolled ones in the field. A known and uniform concentration of pathogenic inoculum allowed us to conclude with high confidence on the effect of the treatments under greenhouse conditions, while the patchy distribution and unknown concentration of *P. brassicae* under field conditions allowed us to describe only the tendency of the effects. However, a high consistency of the results between greenhouse and field experiments was obtained. Under greenhouse conditions, we observed lower efficacy of the treatments as the inoculum of *P. brassicae* increased, agreeing with the report of Narisawa et al. (2005). Likewise, the different levels of the disease and variable efficacy of treatments led us to classify the field plots with three levels of infestation in both sets of field experimentation. Our results suggest that control measures to manage clubroot disease in cruciferous crops should be chosen according to the concentration of *P. brassicae* in the soil. For example, the integrated program in highly infested soils should include the most drastic measures, such as efficient fungicides to reduce the concentration of the pathogen in the soil, large periods of rotation, the use of resistant cultivars, the application of known biological control agents, and additional cultural practices.

Integrated plans to manage the clubroot disease should be evaluated to demonstrate its real reach since pathogen genetics is complex, with wide variation for pathogenicity. Thus, the success of the resistant cultivars depends on the pathogen population present in the soil (Cao et al., 2009; Diederichsen et al., 2009). To our knowledge, there are no studies on the genetic structure of *P. brassicae* populations in Colombia (Gómez, 2017; Botero-Ramírez et al., 2022); however, clubroot is present in all areas where cruciferous crops are grown (Botero-Ramírez et al., 2022). Although the local commercial promoters of resistant cultivars highlight the advantage of genetic control against *P. brassicae*, they also recognize the susceptibility of these plant materials in soils with high pressure of *P. brassicae* inoculum. Our results with the resistant broccoli cv. Monclano suggest that it could be used as an additional tool to manage clubroot, even to reduce the RS concentration in soils with low inoculum pressure, as it was demonstrated by Murakami et al. (2000) with no susceptible cruciferous species, which stimulate the germination of RS.

Although the efficacy of the mixture of Tricotec® (2 g L^−1^) and T2 (20 mL L^−1^) is attributed to T2 instead of Tricotec®, under the uniform and known concentration of *P. brassicae* in the greenhouse, it was interesting how the effects on disease control were more consistent with the combination of Tricotec® and T2 under field conditions.

## Conclusion

The results of this study indicate that the available tools in the local market such as the T2 bacterial consortium and the resistant cultivars may be useful to reduce the losses caused by *P. brassicae* in cruciferous crops. However, the efficacy of available *Trichoderma* spp.-based bioproducts should be tested in future works, since the use of commercial bioproducts is evolving to a usual practice, even more in these production systems where several cropped species and soil-borne phytopathogens converge. Maybe it is necessary to balance the dose of *Trichoderma* according to both the beneficial traits of the strains and the effect desired between plant growth promotion and disease control. These kinds of questions are open and deserve to be answered in the near future. Previous and current evaluations of T2 bioproduct show great potential as a tool to control plant diseases. However, more in-depth research is needed to understand the deployed mechanisms of action against *P. brassicae*, the interactions with the plant host, and the relationship with the native microbiota that leads to effective biocontrol.

## Data availability statement

The raw data supporting the conclusions of this article will be made available by the authors, without undue reservation.

## Author contributions

CM-V: Conceptualization, Data curation, Formal analysis, Investigation, Methodology, Supervision, Writing – original draft, Writing – review & editing. FG-A: Investigation, Validation, Writing – review & editing. LD-M: Investigation, Validation, Writing – review & editing. ER: Investigation, Validation, Writing – review & editing. AV-G: Investigation, Validation, Writing – review & editing. ER-G: Data curation, Investigation, Methodology, Validation, Writing – review & editing. CM-P: Data curation, Investigation, Methodology, Validation, Writing – review & editing. DR-O: Data curation, Investigation, Methodology, Validation, Writing – review & editing. APZ: Investigation, Methodology, Validation, Writing – review & editing.

## References

[B1] Acevedo-Osorio A. Schneider S. (2020). Agricultura Campesina, Familiar y Comunitaria: una perspectiva renovada del campesinado para la construcción de paz en Colombia. Luna Azul 50, 132–156. doi: 10.17151/luaz.2020.50.7

[B2] Ahmed A. Munir S. He P. Li Y. He P. Yixin W.. (2020). Biocontrol arsenals of bacterial endophyte: An imminent triumph against clubroot disease. Microbiol. Res. 241, 126565. doi: 10.1016/j.micres.2020.126565 32829185

[B3] Arie T. Kobayashi Y. Okada G. Kono Y. Yamaguchi I. (1998). Control of soilborne clubroot disease of cruciferous plants by epoxydon from *Phoma glomerata* . Plant Pathol. 47, 743–748. doi: 10.1046/j.1365-3059.1998.00298.x

[B4] Auer S. Ludwig-Müller J. (2023). Biocontrol of clubroot disease: how successful are endophytic fungi and bacteria? Eur. J. Plant Pathol. 167, 433–451. doi: 10.1007/s10658-023-02701-3

[B5] Bhattacharya I. Pramanik M. (1998). Effect of different antagonist rhizobacteria and neem products on clubroot of crucifers. Indian Phytopathol. 51, 87–90.

[B6] Botero-Ramírez A. Gómez I. Benítez E. García C. (2015). Liming with dolomite reduces the efficacy of the biocontrol fungus *Trichoderma koningiopsis* against cabbage clubroot. Agronomía Colombiana 33, 49–57. doi: 10.15446/agron.colomb.v33n1.46759

[B7] Botero-Ramírez A. Padilla-Huertas F. L. Strelkov S. E. García-Domínguez C. (2022). The occurrence of clubroot in Colombia and its relationship with climate and agronomic practices. Horticulturae 8, 711. doi: 10.3390/horticulturae8080711

[B8] Cao T. Manolii V. P. Hwang S. F. Howard R. J. Strelkov S. E. (2009). Virulence and spread of *Plasmodiophora brassicae* (clubroot) in Alberta, Canada. Can. J. Plant Pathol. 31, 321–329. doi: 10.1080/07060660909507606

[B9] Cao T. Manolii V. P. Zhou Q. Hwang S. F. Strelkov S. E. (2019). Effect of canola (*Brassica napus*) cultivar rotation on *Plasmodiophora brassicae* pathotype composition. Can. J. Plant Sci. 100, 218–225. doi: 10.1139/cjps-2019-0126

[B10] Chai A. L. Xie X. W. Shi Y. X. Li B. J. (2014). Research status of clubroot (*Plasmodiophora brassicae*) on cruciferous crops in China. Can. J. Plant Pathol. 36, 142–153. doi: 10.1080/07060661.2013.868829

[B11] Cheah L.-H. Page B. B. C. (1997). *Trichoderma* spp. For potential biocontrol of clubroot of vegetable brassicas. P. NZ. Plan P. 50, 150–153. doi: 10.30843/nzpp.1997.50.11287

[B12] Cheah L.-H. Veerakone S. Kent G. (2000). Biological Control of clubroot on cauliflower with Trichoderma and *Streptomyces* spp. P. NZ. Plan P. 53, 18–21. doi: 10.30843/nzpp.2000.53.3642

[B13] Cruz-Barrera M. Izquierdo-García L. F. Gómez-Marroquín M. Santos-Díaz A. Uribe-Gutiérrez L. Moreno-Velandia C. A. (2022). Hydrogel capsules as a new delivery system for *Trichoderma koningiopsis* Th003 to control *Rhizoctonia solani* in rice (*Oriza sativa*). Res. Square. doi: 10.21203/rs.3.rs-2264585/v1 PMC10894772 38403797

[B14] Cubeta M. A. Porter D. Mozley S. E. (2004). “Laboratory exercises with zoosporic plant pathogens,” in Plant pathology: concepts and laboratory exercises. Eds. Trigiano R. N. Windham M. T. Windham A. S. (New York: CRC Press), 173–192.

[B15] Deora A. Gossen B. McDonald M. (2012). Infection and development of *Plasmodiophora brassicae* in resistant and susceptible canola cultivars. Can. J. Plant Pathol. 34, 239–247. doi: 10.1080/07060661.2012.681071

[B16] Diederichsen E. Frauen M. Linders E. G. A. Hatakeyama K. Hirai M. (2009). Status and perspectives of clubroot resistance breeding in crucifer crops. J. Plant Growth Regul. 28, 265–281. doi: 10.1007/s00344-009-9100-0

[B17] Dixon G. R. (2006). The biology of *Plasmodiophora brassicae* Woronin a review of recent advances. Acta Hortic. 706, 271–282. doi: 10.17660/ActaHortic.2006.706.32

[B18] Dixon G. R. (2014). Clubroot (*Plasmodiophora brassicae* Woronin) – an agricultural and biological challenge worldwide. Can. J. Plant Pathol. 36 (sup1), 5–18. doi: 10.1080/07060661.2013.875487

[B19] El Enshasy H. A. Ambehabati K. K. Hanapi S. Z. Dailin D. J. Malek R. A. (2020). “ *Trichoderma* spp.: A unique fungal biofactory for healthy plant growth,” in Rhizosphere Microbes. Microorganisms for Sustainability, vol 23. Eds. Sharma S. K. Singh U. B. Sahu P. K. Singh H. V. Sharma P. K. (Singapore: Springer), 573–592. doi: 10.1007/978-981-15-9154-9_24

[B20] Elsherif M. Grossman F. (1991). Investigations on biological control of some plant pathogenic fungi by fluorescent pseudomonads using different methods of application. J. Plant Dis. Protect. 98, 236–249.

[B21] EVA (Evaluaciones Agropecuarias Municipales 2019-2021) (2022). Available at: https://www.upra.gov.co/web/guest/evaluaciones-agropecuarias-municipales-eva.

[B22] Fei W. Feng J. Rong S. Strelkov S. E. Gao Z. Hwang S. F. (2016). Infection and gene expression of the clubroot pathogen Plasmodiophora brassicae in resistant and susceptible canola cultivars. Plant Dis. 100, 824–828. doi: 10.1094/PDIS-11-15-1255-RE 30688612

[B23] Gómez S. J. C. (2017). Estimación de las pérdidas en rendimiento ocasionadas por Plasmodiophora brassicae Woronin en cultivos de repollo, brócoli y coliflor (Bogotá, Colombia: National University of Colombia).

[B24] Guo S. Zhang J.-W. Dong L.-H. Xu L. Asif M. Guo O. G.. (2019). Fengycin produced by *Bacillus subtilis* NCD-2 is involved in suppression of clubroot on Chinese cabbage. Biol. Control 136, 104001. doi: 10.1016/j.biocontrol.2019.104001

[B25] He P. Cui W. Munir S. He P. Huang R. Li X.. (2023). Fengycin produced by *Bacillus subtilis* XF-1 plays a major role in the biocontrol of Chinese cabbage clubroot via direct effect and defense stimulation. J. Cell. Physiol. 238, 1–12. doi: 10.1002/jcp.30991 36946428

[B26] ICA (2022) (Instituto Colombiano Agropecuario). Available at: https://www.ica.gov.co/areas/agricola/servicios/agricultura-ecologica-1/documentos/publicacion-bd_rn-rf_-31-mar-2022-1.aspx.

[B27] Izquierdo-García L. F. González-Almario A. Cotes A. M. Moreno-Velandia C. A. (2020). *Trichoderma virens* Gl006 and *Bacillus velezensis* Bs006: a compatible interaction controlling Fusarium wilt of cape gooseberry. Sci. Rep. 10, 6857. doi: 10.1038/s41598-020-63689-y 32321998 PMC7176702

[B28] Kurowski T. P. Majchrzak B. Kowalska E. (2009). The effectiveness of the biological control of clubroot (*Plasmodiophora brassicae*) in Brassicaeae plants. Phytopathologia 52, 5–12.

[B29] Lee S.-O. Choi G.-J. Choi Y.-H. Jang K.-S. Park D.-J. Kim C.-J.. (2008). Isolation and characterization of endophytic Actinomycetes from Chinese cabbage roots as antagonists to *Plasmodiophora brassicae* . J.Microbiol Biotechn. 18, 1741–1746. doi: 10.4014/jmb.0800.108 19047815

[B30] Li X. Y. (2013). Diversity and active mechanism of fengycin type cyclopeptides from *Bacillus subtilis* XF-1 against *Plasmodiophora brassicae* . J. Microbiol. Biotechn. 23, 313–321. doi: 10.4014/jmb.1208.08065 23462003

[B31] Li J. Philp J. Li J. Wei Y. Li H. Yang K.. (2020). *Trichoderma harzianum* inoculation reduces the incidence of clubroot disease in Chinese cabbage by regulating the rhizosphere microbial community. Microorganisms 8, 1325. doi: 10.3390/microorganisms8091325 32878079 PMC7563613

[B32] Liao J. Luo L. Zhang L. Wang L. Shi X. Yang H.. (2022). Comparison of the effects of three fungicides on clubroot disease of tumorous stem mustard and soil bacterial community. J. Soils Sediments 22, 256–271. doi: 10.1007/s11368-021-03073-z

[B33] Ludwig-Müller J. (2016). “Belowground defense strategies against clubroot (*Plasmodiophora brassicae*),” in Belowground Defense Strategies in Plants. Signaling and Communication in Plants. Eds. Vos C. Kazan K. (Cham: Springer), 195–219. doi: 10.1007/978-3-319-42319-7_9

[B34] Mesa P. García C. Cotes A. M. (2017). Searching for an alternative to manage powdery scab caused by *Spongospora subterranea* in potato. Rev. Colombiana Cienc. Hortícolas 11, 378–386. doi: 10.17584/rcch.2017v11i2.6150

[B35] Moreno-Velandia C. A. Castillo F. González A. Bernal D. Jaimes Y. Chaparro M.. (2009). Biological and molecular characterization of the response of tomato plants treated with *Trichoderma koningiopsis.* Physiol. Mol. Plant Pathol. 74, 111–120. doi: 10.1016/j.pmpp.2009.10.001

[B36] Murakami H. Tsushima S. Akimoto T. Murakami K. Goto I. Shishido Y. (2000). Effects of growing leafy daikon (*Raphanus sativus*) on populations of *Plasmodiophora brassicae* (clubroot). Plant Pathol. 49, 584–589. doi: 10.1046/j.1365-3059.2000.00495.x

[B37] Narisawa K. Shimura M. Usuki F. Fukuhara S. Hashiba T. (2005). Effects of pathogen density, soil moisture, and soil pH on biological control of clubroot in Chinese cabbage by *Heteroconium chaetospira* . Plant Dis. 89, 285–290. doi: 10.1094/PD-89-0285 30795351

[B38] Narisawa K. Tokumasu S. Hashiba T. (1998). Suppression of clubroot formation in Chinese cabbage by the root endophytic fungus *Heteroconium chaetospira* . Plant Pathol. 47, 206–210. doi: 10.1046/j.1365-3059.1998.00225.x

[B39] Peng G. Mcgregor L. Lahlali R. Gossen B. Hwang S. Adhikari K.. (2011). Potential biological control of clubroot on canola and crucifer vegetable crops. Plant Pathol. 60, 566–574. doi: 10.1111/j.1365-3059.2010.02400.x

[B40] Rempel C. B. Hutton S. N. Jurke C. J. (2014). Clubroot and the importance of canola in Canada. Can. J. Plant Pathol. 36, 1)19–1)26. doi: 10.1080/07060661.2013.864336

[B41] Saharan G. S. Mehta N. K. Meena P. D. (2021). “The disease: clubroot,” in Clubroot Disease of Crucifers (Singapore: Springer), 47–85. doi: 10.1007/978-981-16-2133-8_2

[B42] Shaner G. Finney R. E. (1977). The effect of nitrogen fertilization on the expression of slow-mildewing resistance in Knox wheat. Phytopathology 67, 1051–1056. doi: 10.1094/Phyto-67-1051 40934422

[B43] Sood M. Kapoor D. Kumar V. Sheteiwy M. S. Ramakrishnan M. Landi M.. (2020). *Trichoderma*: the “ Secrets “ of a multitalented biocontrol agent. Plants 9, 762. doi: 10.3390/plants9060762 32570799 PMC7355703

[B44] Syngenta (2023a) Syngenta Colombia Clapton. Available at: https://www.syngenta.com.co/clapton.

[B45] Syngenta (2023b) Syngenta Colombia clarify. Available at: https://www.syngenta.com.co/clarify (Accessed May 2023).

[B46] Syngenta (2023c) Syngenta Colombia Kilazol. Available at: https://www.syngenta.com.co/kilazol (Accessed May 2023).

[B47] Syngenta (2023d) Syngenta Colombia Tekila. Available at: https://www.syngenta.com.co/tekila (Accessed May 2023).

[B48] Syngenta (2023e) Syngenta Colombia Monclano. Available at: https://www.syngenta.com.co/monclano (Accessed May 2023).

[B49] Torres E. (1972). Reacción de algunas crucíferas al ataque de *Plasmodiophora brassicae* Woronin en Manizales, Colombia. Acta Agronómica 22, 185–207.

[B50] US EPA (UNITED STATES ENVIRONMENTAL PROTECTION AGENCY) (2015) Pesticide Product Label, SERENADE. Available at: https://www3.epa.gov/pesticides/chem_search/ppls/000264-01152-20150803.pdf.

[B51] US EPA (UNITED STATES ENVIRONMENTAL PROTECTION AGENCY) (2021) Pesticide Product Label, PRESTOP. Available at: https://www3.epa.gov/pesticides/chem_search/ppls/064137-00013-20211216.pdf.

[B52] Vargas-Baquero C. D. Cotes A. M. (2023). Microbial consortia as an option for biocontrol of *Stromatinia cepivora* . Can. J. Plant Pathol. doi: 10.1080/07060661.2023.2262959

[B53] Yu X.-X. Zhao Y.-T. Cheng J. Wang W. (2015). Biocontrol effect of *Trichoderma harzianum* T4 on brassica clubroot and analysis of rhizosphere microbial communities based on T-RFLP. Biocontrol Sci. Techn. 25, 1493–1505. doi: 10.1080/09583157.2015.1067762

[B54] Zamani-Noor N. Wallenhammar A.-C. Kaczmarek J. Patar U. R. Zouhar M. Manasova M.. (2022). Pathotype characterization of *Plasmodiophora brassicae*, the cause of clubroot in central Europe and Sweden, (2016–2020). Pathogens 11, 1440. doi: 10.3390/pathogens11121440 36558774 PMC9785528

[B55] Zhu M. He Y. Li Y. Ren T. Liu H. Huang J.. (2020). Two new biocontrol agents against clubroot caused by Plasmodiophora brassicae. Front. Microbiol. 10. doi: 10.3389/fmicb.2019.03099 PMC6986203 32038545

