# Supplementary material for: The potential of PGPR and Trichoderma-based bioproducts and resistant cultivars as tools to manage clubroot disease in cruciferous crops
Source: Front Plant Sci. 2024 Jan 8;14:1323530. doi: 10.3389/fpls.2023.1323530 (PMC10800447; doi:10.3389/fpls.2023.1323530)

Supplementary material

The potential of PGPR and *Trichoderma*-based bioproducts and resistant cultivars as tools to manage clubroot disease in cruciferous crops

Carlos Andrés Moreno-Velandia[1], Francy Liliana Garcia-Arias[1], Lorena Dávila-Mora[1], Edwin Rodríguez[1], Alejandro Villabona-Gélvez[1,2], Eliana Gisela Revelo-Gómez[3], Carlos Alberto Marcillo-Paguay[3], Donald Heberth Riascos-Ortiz[3], Andrea Paola Zuluaga[1]

[1]Centro de Investigación Tibaitatá, Corporación Colombiana de Investigación Agropecuaria (AGROSAVIA), Km 14 vía Bogotá a Mosquera, Mosquera, Colombia. [2] Departamento de Ciencias Biológicas, Facultad de Ciencias, Universidad de los Andes, Cra. 1 #18A-12, Bogotá, Colombia. [3]Centro de Investigación Obonuco, Corporación Colombiana de Investigación Agropecuaria (AGROSAVIA), Km 5 vía Pasto a Obonuco, Pasto, Colombia.

Corresponding author: Carlos Andrés Moreno-Velandia cmoreno@agrosavia.co; Orcid ID: 0000-0001-8692-7613.

Abstract

The objective of this research was to determine the potential use of eco-friendly technologies to reduce the clubroot disease caused by *Plasmodiophora brassicae*, the main constraint of cruciferous crops worldwide. Two commercial bioproducts were evaluated in susceptible broccoli, one based on the PGPR consortium (*Bacillus amyloliquefaciens*, *Bacillus pumilus*, and *Agrobacterium radiobacter* K84) and the other one based on *Trichoderma koningiopsis* Th003 (Tricotec® WG). Additionally, the resistant broccoli cv. Monclano® was tested under two concentrations of resting spores (RS) of *P. brassicae*, 1×10^3 and 1×10^5 RS g−1 of soil. The first phase of evaluations with broccoli was carried out under a greenhouse, while susceptible broccoli, cauliflower, and red cabbage were included in a subsequent field phase. Tebuconazole + Trifloxystrobin mixture and Fluazinam were included as positive controls. The effectiveness of the bioproducts depended on the nature of the biocontrol agent, the concentration of *P. brassicae*, and the dose of treatment. Tricotec® showed consistent plant growth promotion but no biocontrol effect against clubroot, and the rhizobacteria-based bioproduct significantly reduced the disease in both greenhouse and field experiments. Higher disease severity was observed with the higher dose of Tricotec®. Under field conditions, the rhizobacteria reduced the incidence progress by 26%, 39%, and 57% under high, medium, and low pressure of the pathogen, respectively. However, no reduction of clubroot severity under high pressure of the pathogen was observed. Complete inhibition of club formation in roots was achieved via the fungicide, but a phytotoxic effect was observed under greenhouse conditions. Fungicides reduced the incidence progress of clubroot, but not the severity under high inoculum pressure in the field. The fungicides, the bacterial treatment, and the combination of bioproducts tended to delay the progress of the disease compared with the negative control and Tricotec alone. The resistant broccoli showed a low level of disease under high concentrations of *P. brassicae* (less than 10% incidence and up to 2% severity). These results suggested the overall potential of commercial tools based on the PGPR consortium and plant resistance to control *P. brassicae*. The integration of control measures, the role of *Trichoderma* spp. in *P. brassicae*–cruciferous pathosystems, and the need to recover highly infested soils will be discussed.

Figure S1. Broccoli plants treated with Tricotec WG (top photo) and T2 (bottom photo) at two weeks after transplant in soil free of *Plasmodiophora brassicae*. Control: untreated plants.

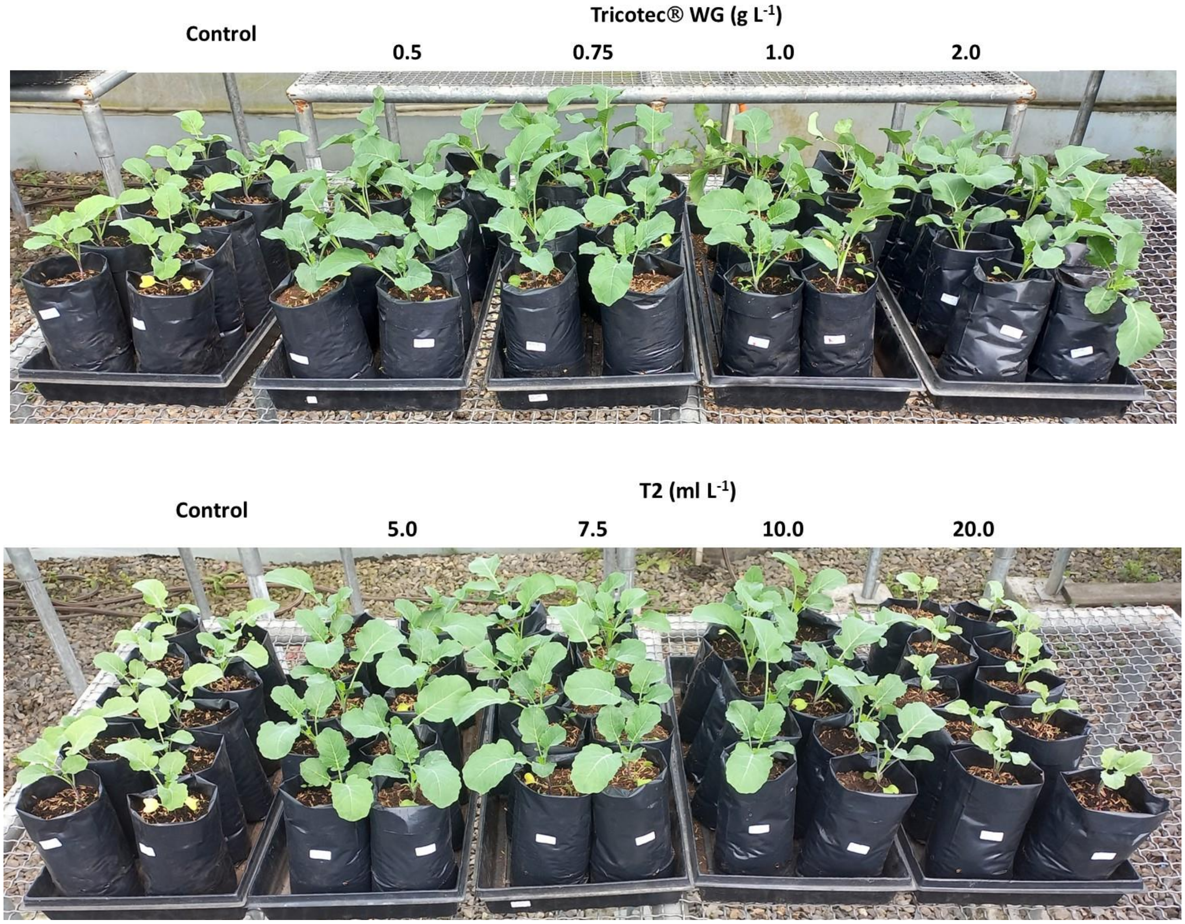

Figure S2. Broccoli plants treated with Tricotec WG and T2 at five weeks after transplant in soil free of *Plasmodiophora brassicae*. Control: untreated plants.

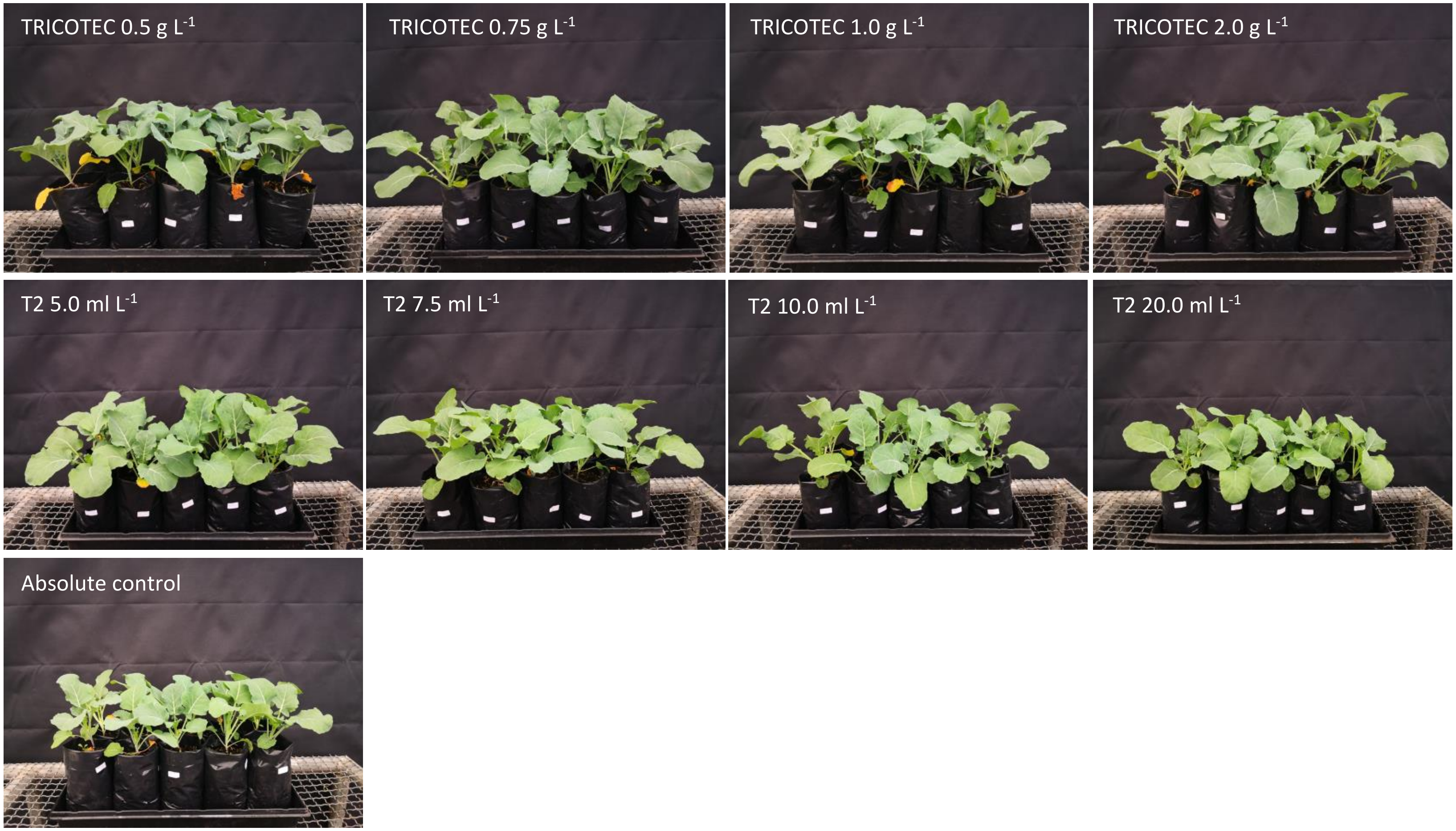

Figure S3. Broccoli plants treated with Tricotec WG (top photo) and T2 (bottom photo) at two weeks after transplant in soil inoculated with *Plasmodiophora brassicae* ($1x10^4$ RS g^{-1}). Absolute control: plants growing in soil free of both *P. brassicae* and treatments against clubroot. Negative control: plants growing in soil inoculated with *P. brassicae*, and untreated against the disease. Positive control: Plants growing in soil inoculated with the pathogen and treated with Nativo®

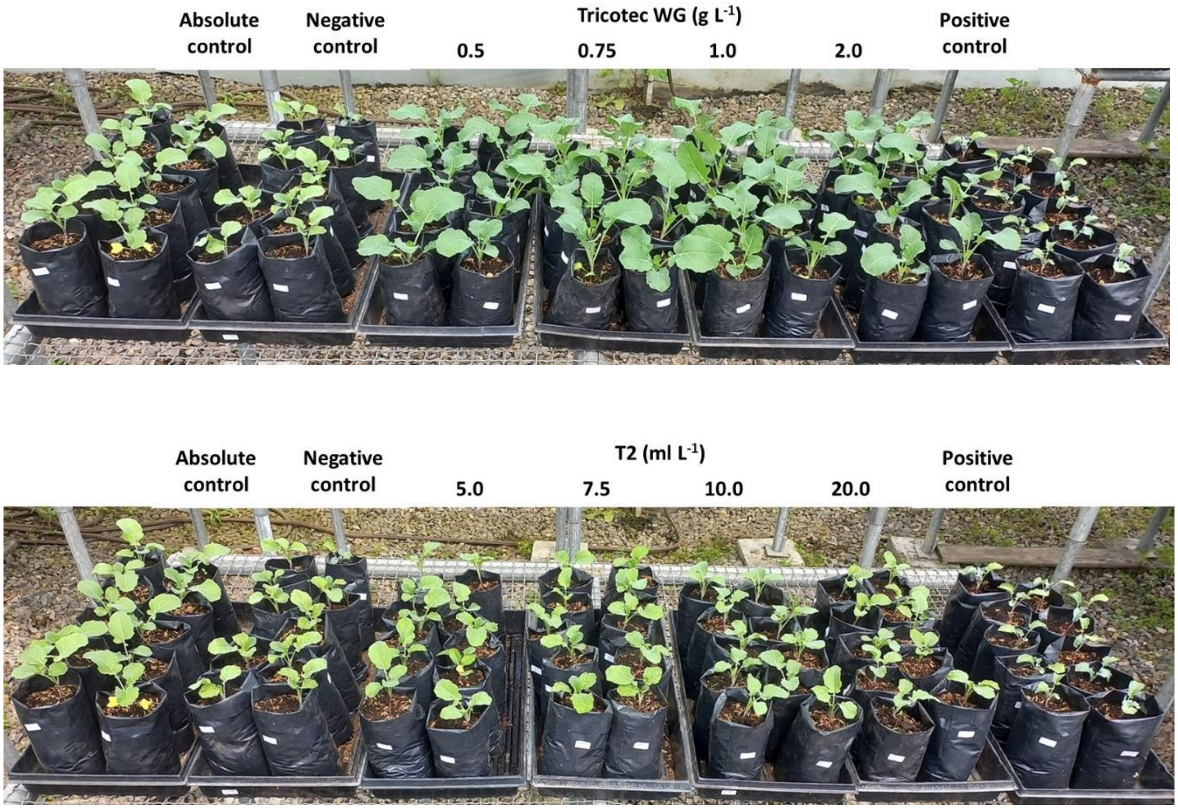

Figure S4. Broccoli plants treated with Tricotec WG and T2 at five weeks after transplant in soil inoculated with *Plasmodiophora brassicae* (Pb - $1x10^4$ RS g^{-1}). Absolute control: plants growing in soil free of both *P. brassicae* and treatments against clubroot. Negative control: plants growing in soil inoculated with *P. brassicae*, and untreated against the disease. Positive control: Plants growing in soil inoculated with the pathogen and treated with Nativo®

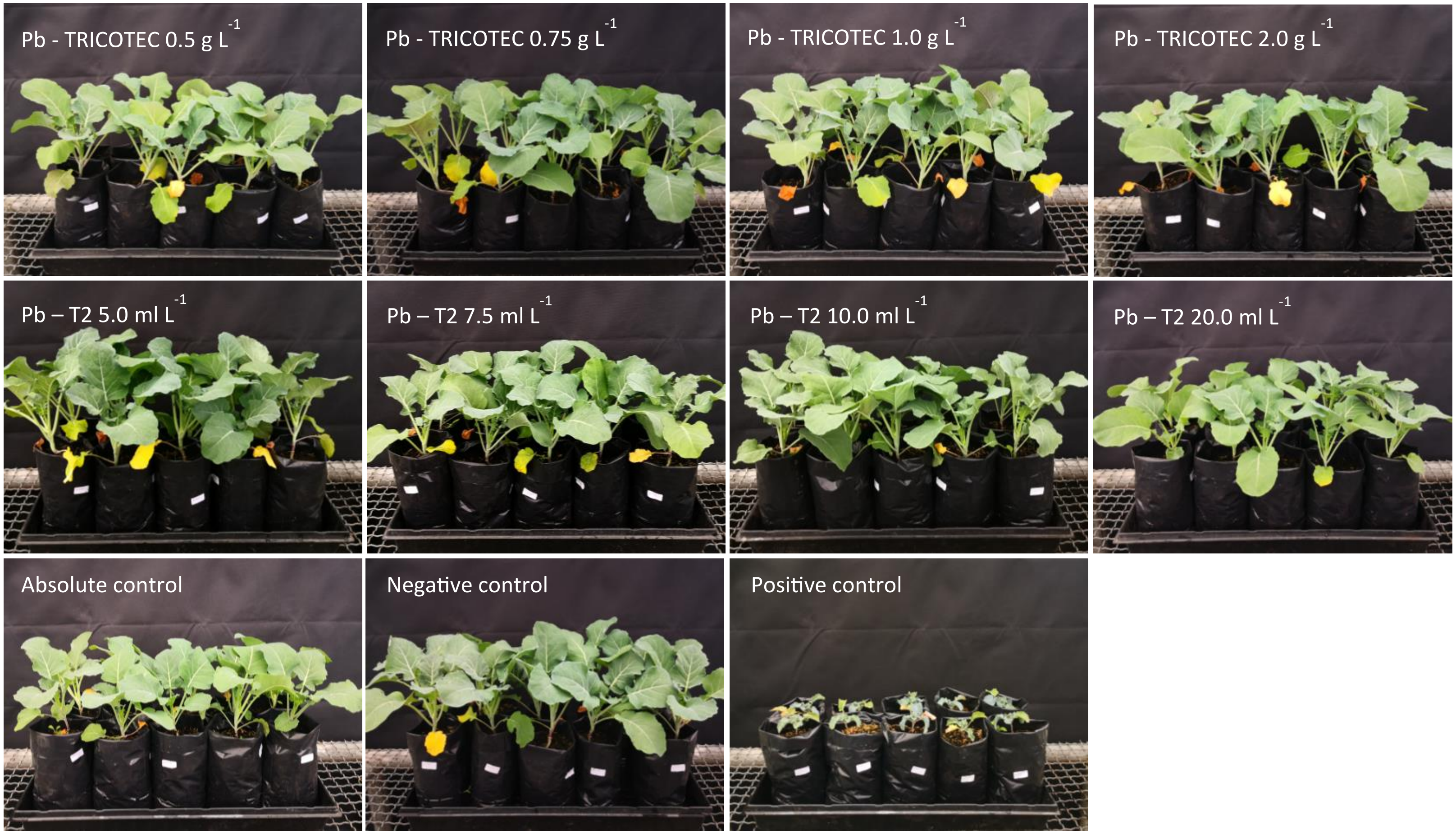

Figure S5. Clubroot incidence progress curves in red cabbage, broccoli, and cauliflower crops under the effect of different doses of the biocontrol treatments Tricotec, T2, and the combination of them in the second set of field experiments. Tested doses of Tricotec were 0.5, 0.75, 1.0, and 2.0 g L^{-1}. Tested doses of T2 were 5.0, 7.5, 10.0, and 20.0 ml L^{-1}. In the negative control (- cntrl) the plants were untreated against clubroot. The positive control (+ cntrl) consisted in drench application of the fungicide Nativo ® (0.6 ml L^{-1}, 30 ml plant-1) at transplant.

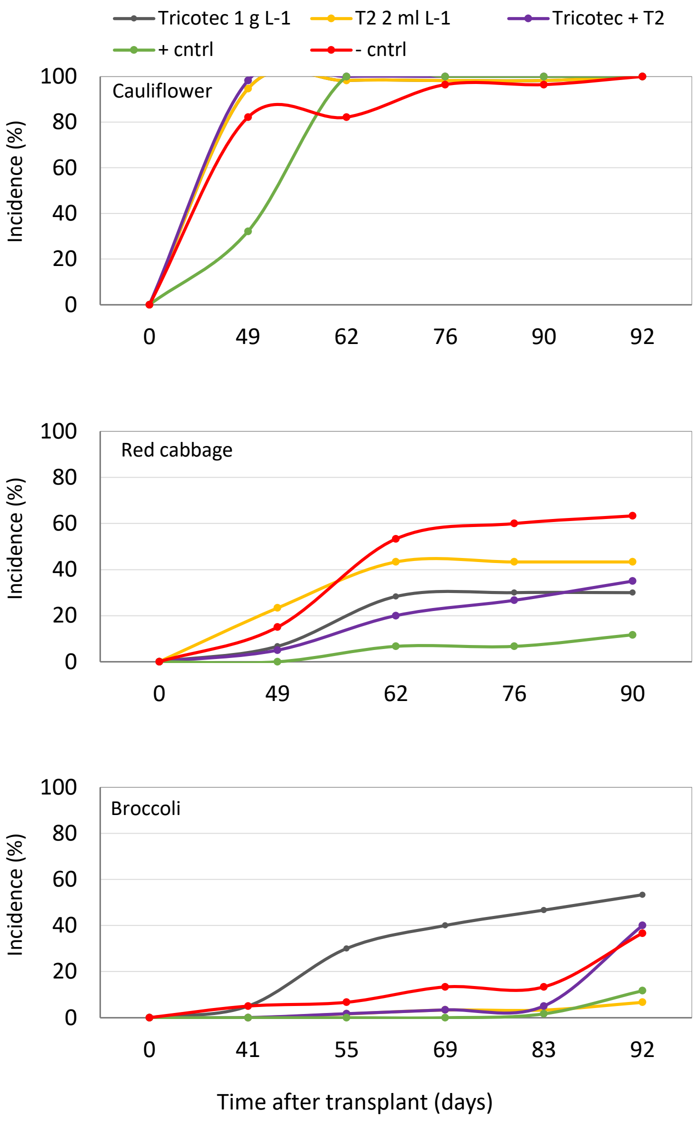

Supplement: Supplementary file 1 [file DataSheet_1.pdf]
